# Art expertise modulates the emotional response to modern art, especially abstract: an ERP investigation

**DOI:** 10.3389/fnhum.2015.00525

**Published:** 2015-09-30

**Authors:** Jane E. Else, Jason Ellis, Elizabeth Orme

**Affiliations:** Department of Psychology, Faculty of Health and Life Sciences, Northumbria UniversityNewcastle upon Tyne, UK

**Keywords:** aesthetics, expertise, ERP, modern art, arousal, attention, abstract art

## Abstract

Art is one of life’s great joys, whether it is beautiful, ugly, sublime or shocking. Aesthetic responses to visual art involve sensory, cognitive and visceral processes. Neuroimaging studies have yielded a wealth of information regarding aesthetic appreciation and beauty using visual art as stimuli, but few have considered the effect of expertise on visual and visceral responses. To study the time course of visual, cognitive and emotional processes in response to visual art we investigated the event-related potentials (ERPs) elicited whilst viewing and rating the visceral affect of three categories of visual art. Two groups, artists and non-artists viewed representational, abstract and indeterminate 20th century art. Early components, particularly the N1, related to attention and effort, and the P2, linked to higher order visual processing, was enhanced for artists when compared to non-artists. This effect was present for all types of art, but further enhanced for abstract art (AA), which was rated as having lowest visceral affect by the non-artists. The later, slow wave processes (500–1000 ms), associated with arousal and sustained attention, also show clear differences between the two groups in response to both type of art and visceral affect. AA increased arousal and sustained attention in artists, whilst it decreased in non-artists. These results suggest that aesthetic response to visual art is affected by both expertise and semantic content.

## Introduction

Art can arouse emotions in many different ways. Great art is as famous for its provocative or shocking content as it is for its beauty and elegance. From Goya’s *Disaster of War* suite of engravings, of the 1800’s, to the Chapman brothers remaking of them in Airfix form in the 1990’s, from Picasso’s powerful and moving anti-war painting, *Guernica* of 1937, to Chris Offilis’s *The Holy Virgin Mary* (1996), with its tension between beauty and ugliness, artists have created a monstrous, hideous, repulsive, wonderful, gorgeous and often beautiful, world. Whilst aesthetics was the dominant movement in art *until* the 20th century, modern art *of* the 20th century has changed both what we think of as art and how we think about art. It has been argued that all various “isms” of modern art seem to be departures from beauty, in fact “anti-loveliness” seems to be more the norm (Collings, 1999). In recent years using words like “quality” or “beauty” about art can be interpreted as amateurish enthusiasm rather than knowledgeable connoisseurship (Meecham and Sheldon, 2005). Whilst they have not disappeared altogether from modern art they are no longer central to the appreciation of art (Collings, 1999).

Although aesthetic appraisal may not always be about beauty Scruton (2009) argues that beauty in its general sense is generally the subject matter of aesthetic judgement. Much of the imaging research in the realm of neuroaesthetics (Cela-Conde et al., 2004, 2011; Chatterjee, 2004, 2011; Leder et al., 2004; Cupchik et al., 2009; Skov and Vartanian, 2009), exploring aesthetic judgement (Jacobsen and Hofel, 2001; Nadal et al., 2008), aesthetic processing (Kirk, 2008; Kirk et al., 2009a,b), preference (Hansen et al., 2000; Kawabata and Zeki, 2004; Vartanian and Goel, 2004a,b), or beauty (Zeki, 1999, 2001, 2002) appears to follow the traditional view, accepted from Plato until Kant and beyond, that beauty be considered the paradigmatic aesthetic quality. Research regarding visual art has also tended to focus on neural responses to beauty (e.g., Kawabata and Zeki, 2004; de Tommaso et al., 2008), rather than reactions to particular features of an artwork (e.g., Salimpoor et al., 2011) and employed stimuli that generated agreement regarding beauty, or lack of it (e.g., Kawabata and Zeki, 2004; de Tommaso et al., 2008). Few have explored variation in the degree of arousal rather than response to beauty and preference (e.g., Vessel et al., 2012), or unusual emotions such as confusion, contempt, surprise or embarrassment (Silvia, 2009).

In order to test whether aesthetic affect depends on meaningful content Ishai et al. (2007) classified art into three categories. Indeterminate art (IA), in which objects are ephemeral, invokes a perceptual conundrum, apparently detailed and vivid images remain unrecognised (Pepperell, 2006). Alternatively, representational or figurative art represents reality in a straightforward way. Yet it is more than a view into reality, its intention is to evoke subjective reactions to stylistic and structural properties. In contrast, abstract art (AA) depicts neither natural forms nor objects, but uses line, color and shape to evoke emotional and aesthetic responses. As such, comparing the emotional effect of representational art (RA) directly with AA may create difficulties in interpretation. The effect reported may simply be in response to semantic content, rather than art. It may be difficult to differentiate between emotions evoked by subject matter (i.e., faces, animals, landscapes), rather than its interpretation by the artist, or viewer. Although Ishai et al. (2007) found little difference in affect ratings, more representational than indeterminate paintings were remembered, and affective strength increased the probability of subsequent recall. However, here all the indeterminate artworks were by one artist (Robert Pepperell), whereas the representational paintings were by various artists. It could be argued that the enhanced memory and affect was due to greater variety of the art. Nevertheless, their results suggest that whilst aesthetic affect appears to depend on formal visual features, perception and memory of art depend on semantic aspects.

Models of aesthetic processing proposed by Chatterjee (2004) and Leder et al. (2004) posit that the emotional response to art is what distinguishes the aesthetic response from responses to other visual stimuli, and that it involves not only perception but memory, cognitive mastering (measured by level of expertise), evaluation and knowledge. Whereas Chatterjee (2004) and Leder et al. (2004) associate this response with liking and wanting, or pleasure, Silvia (2009) acknowledges that modern art may also cause confusion, requiring more attention or effort to process. By comparing the responses to three different types of modern art, abstract, representational and indeterminate, we hope to be able to differentiate those responses influenced by semantic content with those influenced by other visual features of art, and to widen the range of emotions evoked.

Interestingly, Silvia (2006) found that whilst art experts find art more interesting and understandable, particularly complex or AA, people high and low in art training make the same emotional appraisals of art. Despite extensive research into the neural correlates of the effect of expertise in the music and auditory domain (e.g., Besson and Faita, 1995; Koelsch et al., 2002; Bhattacharya and Petsche, 2005; Tervaniemi et al., 2009), there has been relatively little research into the effect of expertise on visual perception of art. The limited research that does exist has revealed differences in brain activity, specifically in the reward related areas, whilst performing art-related tasks (Solso, 2001; Bhattacharya and Petsche, 2002), whilst making aesthetic judgements (Kirk et al., 2009a), and during the contemplation of art (Pang et al., 2013). Differences between art experts and non-experts in various aspects of aesthetic preference and judgements (e.g., Smith and Melara, 1990; Hekkert and van Wieringen, 1996; Furnham and Walker, 2001), in methods of processing complexity (Reber et al., 2004), how they view and perceive pictures (Vogt and Magnusson, 2007) have been identified. A relevant question to consider, therefore, is whether formal art training impacts on the various, complex cognitive, evaluative and affective processes involved in the contemplation of art. Whilst previous event-related potential (ERP) research suggests that artists should demonstrate an enhanced response to arousing artworks due to their increased attention to and memory for art, Pang et al. (2013) propose the opposite, expertise reduces the response due to increased neural efficiency. As such, it will be interesting to explore the effect of expertise on visceral responses to modern art, in addition to the underlying neural activity.

The ERP technique provides a methodology that can directly measure the neural events in response to visual art with high temporal precision. ERP’s can also “covertly” assess cognitive responses when overt behavioral responses cannot be reliably obtained, or can be used to record disassociations between ERP activity and behavioral responses (Luck, 2012). The oddball paradigm is often used in the investigation of ERP components due to its success in evoking robust and reliable markers of cognitive function (Huettel and McCarthy, 2004), particularly the P3 component. Here, we utilized the oddball paradigm in order to identify differences in neural responses both to the three categories of modern visual art and simple non target stimuli, and also between the different art types.

ERP studies of emotion, typically using affective pictures as stimuli (for a review, see Olofsson et al., 2008) have identified the five components sensitive to emotional content and the time course of emotional processing of the stimuli as P1, N1/N170, P2, N2 and P3 (Hajcak et al., 2012). The focus of research has often been on two emotional components, first the *early posterior negativity* (EPN), a negative potential over the visual cortex in the N2 latency range, and secondly, the *late positive potential* (LPP), a positive potential that usually has the same onset time and scalp distribution as the P3 component, but which may extend over many hundreds of milliseconds (Luck, 2012). Subsequently, the ERP components of interest in this study are the exogenous P1, N1, P2, N2, and the endogenous P3 and LPP.

The P1 appears to be influenced by attention and arousal (Hillyard et al., 1998; Vogel and Luck, 2000) and is sensitive to a number of early visual perception inputs including luminance and contrast (Bradley et al., 2007). The visual N1, although widely distributed over the scalp, peaks earlier over frontal than posterior sites. It is indexed to the allocation of attentional resources, which influence the selection and discrimination of perceptual features such as color, luminance or motion (Anllo-Vento and Hillyard, 1996; Vogel and Luck, 2000). The N170, typically peaking around 170 ms after stimulus onset, is a robust and frequently reported component associated with face processing and recognition research (Rossion and Jacques, 2008). However, it is also consistent with expertise, with an enhanced N170 reported in response to dogs, in dog experts, birds, in bird experts, and fingerprints, in fingerprint experts (Tanaka and Curran, 2001; Busey and Vanderkolk, 2005, as cited in Luck, 2012), and perhaps to art, in art experts. Although little is known about the P2 component it is associated with higher level perceptual and attentional processing of visual stimuli (Luck and Hillyard, 1994; Hajcak et al., 2012), and has shown greater amplitudes in response to emotional stimuli and affective pictures, than to neutral (Carretié et al., 2001, 2004; Delplanque et al., 2004; Olofsson and Polich, 2007). Due to the artists’ interest and knowledge we expect to see larger amplitude of the P1 and P2 at occipito-parietal sites, and N1 at frontal sites, in response to all categories of art, reflecting increased attentional resources and discrimination of the perceptual features, compared to the non-artists.

The N2, a central negativity, peaking 250 ms after stimuli presentation (Carretié et al., 2004), appears to index selective attention to specific stimulus features (e.g., color, shape and form; Codispoti et al., 2006), and to reflect the actual process of categorizing the stimulus (Luck, 2012). Both emotional and neutral stimuli have been shown to influence the magnitude of the N2, although it is not clear whether this effect is equal for both pleasant and unpleasant stimuli (e.g., Carretié et al., 2004; de Tommaso et al., 2008). It has previously been explored with regard to the processing of style and content in visual art (Augustin et al., 2011), with the onset of the potential appearing to be slightly later in response to style than to content. Increased amplitude of the N2 in response to neutral rather than to beautiful pictures has been attributed to difficulty of discrimination (de Tommaso et al., 2008). Due to the content and emotional salience we expect to see this effect in response to indeterminate and RA, at central sites, as opposed to abstract, in both groups (Codispoti et al., 2006; Dell’acqua et al., 2010), reflecting increased attention, recognition processes and categorization (Luck, 2012).

In studies dating back over 50 years, emotional images compared to neutral elicit an increased positivity 300–500 ms after stimuli presentation (e.g., Lifshitz, 1966, as cited in Hajcak et al., 2012). The P3, a positive deflection peaking approximately 300–350 ms post-stimulus, is observed at central frontal and parietal sites. It is most often observed in the oddball paradigm, with oddball stimuli eliciting a much larger P3 than standard stimuli. The late part of this affective ERP (>300 ms) is dominated by the P3 wave and the following positive slow wave. The distinguishing feature is its sensitivity to target probability (Luck, 2005); it only occurs if the subject is actively engaged in the task of detecting the stimuli. It appears to be sensitive to motivational significance, is heavily dependent on attention (Hajcak et al., 2012), task relevance, and arousal (Duncan-Johnson and Donchin, 1977; Polich, 2007). It is also linked to context updating in working memory (Donchin and Coles, 1988), knowledge in long-term memory (Leder et al., 2004; Polich, 2007) categorization (Luck, 2012), and has been reported to be sensitive to beauty and aesthetic discrimination of artworks (de Tommaso et al., 2008). The LPP has also been correlated with subjective arousal ratings, suggesting it mirrors subjective emotional experience (Luck, 2012). Pang et al. (2013) attribute an association between reduced “higher order” ERP amplitudes (P3b and LPC) and art-expertise under free viewing of art and non-art stimuli, to increased neural efficiency; the appreciation of visual art relies on cognitive and neural processes involved in processing other visual stimuli. In contradiction to this we expect to find increased amplitude of the P3 and LPP in artists in response to all three categories of art, reflecting not only directed attention during the task (Luck, 2005; Hajcak et al., 2012), but also their interest and knowledge (Donchin and Coles, 1988; Leder et al., 2004; Polich, 2007) and enhanced arousal response to art (Duncan-Johnson and Donchin, 1977; Polich, 2007).

In summary, whilst cognitive and affective processes involved in aesthetic experience, judgement, processing and preference have been explored, little is known about the wider range of emotional responses elicited by art. If art is about eliciting an emotional response (Solso, 2003) it is not *necessarily* about beauty. Aesthetic experiences may be focussed on conceptual or perceptual features (Shimamura and Shimamura, 2012), and on unusual and negative emotions (Silvia, 2009). Feelings of shock, ugliness, disgust and disinterest are as likely to be provoked as those associated with wonder, beauty and fascination. In light of this it is the aim of this EEG study to focus on raw visceral and emotional responses during the perception of art, rather than the judgement of beauty.

With this in mind the experiment was conducted with four aims in mind; to examine how affective responses to modern art impacts on emotion/arousal and behavioral measures of cognitive performance; to examine the moderating effects of category of art (abstract, representational and indeterminate) on these measures; to examine endogenous and exogenous ERP components elicited by an oddball paradigm to investigate the perceptual and emotional response to the art, and finally, to examine the moderating effect of expertise on these points. It is expected that expertise will positively influence the rating of affect, particularly with regard to AA. It is reasonable to assume that both groups will demonstrate greater ERP amplitude in response to representational and IA than to abstract, due to the actual or perceived semantic content, and that artists will show larger magnitude than non-artists to all categories of art, but with a more pronounced difference in response to AA.

## Materials and Methods

### Participants

A total of 36 right-handed participants consented and took part in the study. Nineteen were non-artists (five males). Seventeen were artists (five males). Artists were classified as such if they identified themselves as a visual artist, had more than 3 years higher education in Fine Art, and were working in the visual art domain at the time of the study, e.g., as an artist, art historian, curator, advisor. Any potential participants who identified themselves as visual artists, but did not meet these criteria were excluded from taking any further part in the study. Similarly, those who met the criteria for an artist, but did not identify themselves as such were also excluded. Artists reported visiting art galleries more than six times* per annum*, whereas non-artists reported fewer than three visits* per annum*. All were recruited from the Universities of Northumbria and Newcastle staff and undergraduate or graduate populations. Artists were also recruited from artist networks. All were right handed, fluent English speakers and reported normal or corrected to normal vision and no history of neurological damage. The study received ethical approval from the School of Life Sciences Ethics Committee at Northumbria University and was conducted in accordance with the Declaration of Helsinki. All participants gave their written informed consent before inclusion in the study and were paid or given course credits for their participation. Further participant information is given in Table 1.

**Table 1:** **Mean (and *SD*) participant characteristics, showing gender, age, years of further education, years of further art education, national adult reading test and toronto alexythimia scale scores**.

	Artists (A)	Non-artists (NA)	*t*_(34)_	*p*
Gender F:M	12:5	14:5		
Age (years)	26.6 (5)	22.1 (3.1)	2.7	*p* < 0.05
Education (years)	19.1 (2.5)	16.6 (2.4)	2.94	*p* < 0.01
Art education (years)	5.6 (1.4)	0.1 (0.7)	16.45	*p* < 0.001
NART	35.8 (5.3)	31 (6.8)	2.04	*p* < 0.05
TAS-20	46.6 (11.3)	47.2 (14.4)	15	*p* = 0.88

### Stimuli

A pilot study was conducted to identify the target art stimuli. The artworks selected were all by artists from The Times Top 200 Artists of the 20th Century to Now (2009), a poll run in conjunction with Saatchi Gallery. This poll was used in an attempt to eliminate personal bias and to ensure a wide range of artists, styles, schools of art, subject matter and medium. Thirty-three artists on the list were not included for two reasons: (i) they are sculptors, and (ii) no work is accessible in national collections. For ethical reasons, images that depicted *extreme* violence or horror or which contained *strong* religious or sexually explicit imagery were also avoided. All works selected were two-dimensional images to ensure accurate reproduction on the computer screen. Paintings, drawings, mixed media and photographs, portraits, animals, landscapes, abstract shapes and forms were all included. High quality jpeg files of the pictures were downloaded from the online collections of the National Galleries of England and Scotland, Museum of Modern Art (MoMA), TATE, or by kind permission of the artist (David Hockney, Inc.). This ensured not only the highest quality possible images of the artworks, but also acknowledged they were representative of the artist and worthy of a national collection. In order to reproduce the artworks on a computer screen all were resized to fit within a 730 × 730 pixel format, with a resolution of 96 dpi. Whilst this resulted in changes to the scale of the images (the scale of the originals ranged from 303 × 378 mm to 3136 × 2254 mm), this was the only adjustment made in an effort to retain the integrity of the image. No changes were made to the original color or luminance. Graphic manipulation of the stimuli was done using Paint.NET v3.5.8. The pilot study required participants to categorize 450 artworks into one of three categories, abstract, indeterminate or RA. AA was defined as containing “simple forms, not related to anything” TATE, (2010), RA as “represents reality in a straightforward way, retains strong references to the real world” TATE, (2010), and IA as “highly suggestive of forms but not exactly descriptive of them” (Fairhall and Ishai, 2008). From this categorization 100 artworks in each category were identified, AA, *n* = 100, RA, *n* = 100, and IA, *n* = 100. Seventy five RA, approximately (due to the nature of the art) 50 IA and no AA contained faces or figures (*For a full catalog of the artworks selected see Supplementary Material*). The non-target frequent (FSS) stimulus (*n* = 1800) was a green square and the non-target rare (RSS) stimulus (*n* = 300) was a red circle. Both non-target stimuli were 397 × 397 mm, 150 × 150 pixels. All stimuli were presented in the center of the screen on a white background.

### Materials

#### National Adult Reading Test (NART)

A brief vocabulary test usually used as a measure of premorbid intellectual ability, but which also provides a valid estimate of WAIS IQ (Weschler Adult Intelligence Scale; Crawford et al., 1989). Participants read 50 short words with irregular pronunciation out loud and are given a point for each word pronounced correctly, according to Collins English Dictionary conventions (Coltheart et al., 1987).

#### Toronto Alexithymia Scale (TAS-20)

A widely used 20-item, self-report instrument, with a five-point Likert rating format. It is often used as a measure of alexithymia, or emotional intelligence (Parker et al., 2001). The TAS- 20 consists of three factors: difficulty identifying feelings (Factor 1), difficulty describing feelings (Factor 2), and externally-oriented thinking (Factor 3).

#### Familiarization and Oddball Task

**Figure 1:**
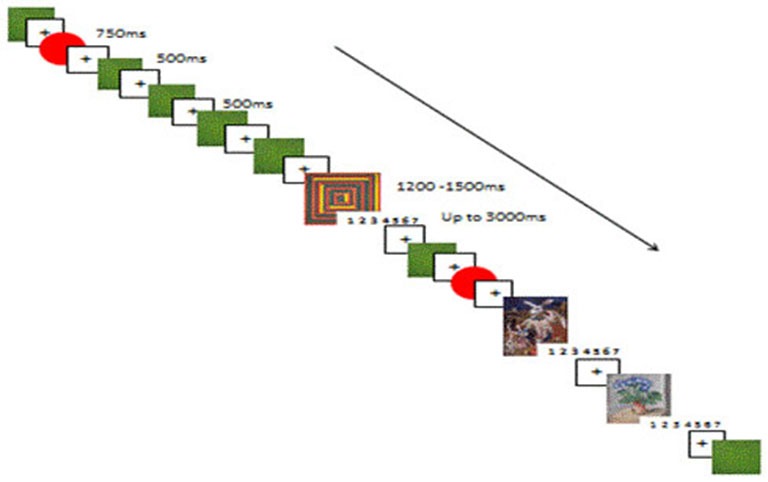
**A reproduction of the experimental paradigm, showing the succession of target (1200–1500 ms), frequent (500 ms) and rare (750 ms) non-target stimuli, and fixation cross (500 ms).** After each target stimuli the rating screen appeared for up to 3000 ms, during which time participants had to give the target stimuli a visceral affective rating (from 1–7).

The task was conducted in five blocks. The first was a nine trial familiarization block, followed by four experimental oddball blocks. See Figure 1 for a reproduction of the experimental paradigm. After each block there was an opportunity for a break. Each experimental block contained 75 art stimuli, 75 rating screens, 75 rare simple stimuli; red circles (RSS), and 450 frequent simple stimuli; green squares (FSS). All stimuli were presented randomly, and each block was presented randomly to each participant. Before each stimulus a fixation cross appeared for 500 ms; the non-target FSS were presented for 500 ms, the non-target RSS were presented for 750 ms and the target stimuli (art) were presented for between 1200–1500 ms. Each target stimulus was followed by a rating screen presented for up to 3000 ms. The rating screen disappeared as soon as the participant responded. The choice of presentation time of the target stimuli of between 1200 and 1500 ms, and 3000 ms for the response screen was based on the mean response times for classification of categories of art in the Pilot Study (990–1360 ms). The probability of the target/rare stimuli to the frequent stimuli was 12.5%:- 75%. Each stimulus was preceded by a black centered fixation cross. Each target stimuli was followed by an affect rating screen, with a scale of 1–7, with 1 = no affect and 7 = lots of affect.

### Apparatus

Testing took place in the EEG lab at Northumbria University. The oddball task was presented using E-Prime^™^ presentation software (E-Prime 2.0, Psychology Software Tools) on a Windows desktop PC, 17 1/2-inch color monitor. EEG was recorded using the ActiView acquisition programme and the Biosemi Active Two, multi-channel, high resolution measurement system. BDF file-format conversion to Neuroscan CNT file-format was converted using Polyrex (Kayser, 2003). ERP averaging was carried out off-line using Neuroscan SCAN 4.3 software.

### EEG/ERP Recording and Data Reduction

EEGs were recorded from 32 electrodes mounted on an elastic electrode cap (Biosemi, Amsterdam, Netherlands) based on the extended international 10–20 system (Jasper, 1958). The montage included four midline sites (Fz, Cz, Pz, Oz), 14 sites over the left hemisphere (FP1, AF3, F3, F7, FC1, FC5, C3, T7, CP1, CP5, P3, P7, PO3, O1), and 14 sites over the right hemisphere (FP2, AF4, F4, F8, FC2, FC6, C4, T8, CP2, CP6, P4, P8, PO4, O2). All EEG recordings were referenced to linked mastoid processes, reference electrodes were placed on the left and right mastoid. To assess eye blink movement, electrodes were placed above and below the right eye to record the vertical electrooculogram (EOG). All signals were digitized at a rate of 2048 Hz, with a recording epoch of −200 to 1400 ms and bandpass filtered between 0.5 and 30 Hz for offline analysis. Automatic eye blink correction, artifact rejection (values outside the range of −75 μV to +75 μV), and ERP averaging were carried out off-line. Target trials with no behavioral response in the interval of 100–1500 ms were excluded.

Prior to data analysis, the data from six participants (four non-artists, two artists, all female) were discarded due to technical difficulties during data acquisition. Data from four participants (two non-artists, one male, two female artists) were discarded because of excessive artifacts found in the EEG data. Therefore the total number of participants included in each of the grand averages for encoding was 13 artists (five males) and 13 non-artists (four males). After eye-blink correction and removal of trials with artifacts, the remaining trials were used in the analysis of each group’s responses to the stimuli. A category for individual participants was rejected for averaging if the number of artifact free trials was less than 16 per stimuli. ERP waveforms were created through averaging EEG data for each of the five stimuli, non-target frequent stimuli (FSS), non-target rare stimuli (RSS), AA, RA and IA, for each group. See Table 2 for the total (*and mean*) number of artifact free accepted trials analyzed for each group; artists and non-artists, and for each stimuli; FSS, RSS, AA, RA and IA. Within target stimulus type, the number of accepted trials *(and mean)* was further subdivided according to level of affect.

**Table 2:** **Total (*and mean*) number of artifact free accepted trials analyzed for each group; Artists (A) and Non-Artists (NA) and for each stimuli; frequent standard stimuli (FSS), rare standard stimuli (RSS), abstract art (AA), representational art (RA) and indeterminate art (IA)**.

Stimuli	Frequent standard stimuli (FSS)	Rare standard stimuli (RSS)	Abstract art (AA)	Representational art (RA)	Indeterminate art (IA)	
Artists (A)	23309 *(1793)*	3821 *(294)*	1249 *(96)*	1285 *(99)*	1257 *(97)*	
Non-artists (NA)	24930 *(1918)*	4164 *(320)*	1407 *(108)*	1411 *(109)*	1390 *(107)*	

**Stimuli**	**Abstract art (AA)**		**Representational art (RA)**		**Indeterminate art (AA)**	
**Level of affect**	**Low affect**	**High affect**	**Low affect**	**High affect**	**Low affect**	**High affect**

Artists (A)	491 *(38)*	513 *(39)*	508 *(39)*	542 *(42)*	411 *(32)*	617 *(47)*
Non-artists (NA)	775 *(60)*	419 *(32)*	430 *(33)*	667 *(51)*	407 *(31)*	657 *(51)*

Within stimulus type, the number of accepted trials *(and mean)* is then further subdivided according to level of affect; low or high affect.

ERP analyses were conducted on mean amplitude and latency values for specific sets of electrodes within specific time windows. These narrow time windows were selected by visual inspection of the grand average ERPs in each group and by predefinition from previous studies in the literature on visually evoked ERPS (see Luck, 2005, p. 34, for a summary of ERP components, and Olofsson et al., 2008 for a review of ERP components elicited in response to affective pictures). Mean amplitude was defined as the average deflection occurring within the selected interval and the mean latency was defined as the time point at which the deflection reached its maximum amplitude. See Table 3 for the time windows selected for the non-target frequent and rare standard stimuli (FSS and RSS) and for the target stimuli (AA, RA, IA). The rationale was that the ERP components elicited in response to passive, non-target FSS and RSS will provide a *baseline* comparison for our investigation of unconscious and conscious components evoked in response to active, target art stimuli (Bennington and Polich, 1999; Huettel and McCarthy, 2004). The oddball procedure was used to minimise habituation effects, to ensure any differences in ERP amplitude and latency were due to differences in target stimuli qualities. To allow for the analysis of both hemisphere and region, 12 electrodes were selected for analysis; F3, Fz, F4, C3, Cz, C4, P3, Pz, P4, O1, Oz and O2.

**Table 3:** **Time windows (in milliseconds) selected for ERP components P1, N1, P2, N2, P3 and LPP in response to non-target frequent and rare stimuli (FSS, RSS), and to target stimuli, abstract, representational and indeterminate art (AA, RA, IA)**.

Stimuli	*P1*	*N1*	*P2*	*N2*	*P3*	LPP
FSS, RSS	100–140 ms	130–150 ms	150–220 ms	150–250 ms	250–500 ms	
AA, RA, IA	100–180 ms	170–220 ms	190–330 ms	275–350 ms	340–590 ms	500–1000 ms

### Procedure

A short questionnaire gathering information regarding demographics, handedness, any problems with vision, previous brain injuries, years of further education, years of art education and number of visits to art galleries* per annum*, was first completed. The national adult reading test (NART) and TAS-20 were then administered to identify any differences in general IQ and emotional intelligence. The electrode cap was fitted and participants were seated in a comfortable chair in front of the monitor at a distance of 90 cm, with the keyboard directly in front. The experimenter then briefed participants regarding the experiment protocol. Participants were requested to move as little as possible and to try not to chew or blink during the experiment blocks. They were told they were going to see three stimuli on the screen: green squares, red circles and pictures of art. Before each of these stimuli they would see a blank screen with a + in the middle. They were asked to focus on the + in preparation for the next stimuli. After each picture of art there would be a rating screen asking them to rate the picture just seen regarding how much it “affected” them, on a scale of 1–7, with 1 = not at all and 7 = a lot. Participants were instructed to use the numbers at the top of the keyboard and to make their decision regarding the level of affect of the picture as quickly as possible. They were instructed to rate their level of affect regarding how much the picture moved them, either positively or negatively, how much “WOW” it had. “Affect” was described as their immediate, visceral, emotional, intuitive response, to the picture, not necessarily how beautiful, good, pretty, ugly, colorful, shocking or famous they thought it was. On completion of the experiment the electrode cap removed and the participant was debriefed. Total EEG recording time was approximately 80 min, with four self-paced rest periods. Total time in the lab was about 2 h.

### Analysis

A 2 (Group; artist and non-artist) × 3 (target type; art, frequent non-target and infrequent non-target) × 3 (category; abstract, representational, IA) mixed design was employed. The between subject factor was group (artist and non-artist). The within-subjects factors were stimulus type and category of art. Measurements of rating of affect (1–7) and time taken by the participant to provide the rating were taken. Measurement of reaction time (RT) was measured in milliseconds (ms) from stimulus onset to button press response. The mean frequency of pictures rated for low and high affect judgements was calculated separately. Low affect was categorised as those with a rating of 1–3, whilst high affect was categorised as having a rating of 5–7. Those with a rating of 4 were excluded from further analysis.

For data reduction purposes and to address the research questions only main and interaction effects for the factors of group and stimulus type were considered.

For analysis of the rating of the level of affect for each category of art, AA, RA and IA, and the response time for the rating of the level of affect for each category of art, for the two groups, a 2 (group: artist and non-artist) × 3 (category of art: AA, RA, IA) analysis of variance (ANOVA) was calculated for the mean level of affect and response time, and also for the frequency of rating for high and low affect judgements and response times.

Prior to the analysis of the specific ERP components *t*-tests confirmed that the number of trials missed by participants (i.e., when they did not respond, or when the data was rejected due to artifacts) did not significantly differ between the groups for each category of art.

Analysis was conducted on the ERP components evoked in response to the non-target frequent and rare stimuli, but is not included in this paper *(See Table 5a in Supplementary Material which outlines all significant main effects and interactions)*.

For the target art stimuli, the mean amplitude and latency of each ERP component was analyzed in a 2 (Group: artist and non-artist) × 3 (Stimuli: AA, RA, IA) × 3 (Hemisphere: Left, Mid line, Right) × 4 (Location: Frontal, Central, Parietal, Occipital) ANOVA.

Initial analyses of covariance were carried out on the data to investigate the moderating effects of age, years of education, and vocabulary ability. *Post hoc* analyses were Bonferroni corrected to adjust for multiple comparisons, with a significance level of *p* < 0.05 unless otherwise stated. In instances where the data failed the sphericity test (*p* < 0.05) the Greenhouse-Geisser corrected degrees of freedom were employed.

## Results

### Behavioral Results

#### Analysis of Rating of Level of Affect and Response Times (RT) for Each Category of Art

See Table 4 for the mean (and SD) rating of the level of affect, mean RT (in ms) for rating the level of affect, and mean frequency of ratings of low affect and high affect for three categories of art, AA, RA, IA and two groups non-artists (NA), and artists (A).

**Table 4:** **Mean (and SD) rating of the level of affect, mean response latency (in ms) for rating the level of affect, and mean frequency of ratings of low affect and high affect for three categories of art, abstract art (AA) representational art (RA), indeterminate art (IA) and 2 groups non-artist (NA) and artist (A)**.

	Mean rating of level of affect	Mean response latency (ms)	Mean frequency of rating of low affect	Mean frequency of rating of high affect
*Artists (A)*				
Abstract art (AA)	3.97 *(0.68)*	1128 *(373)*	39.47 *(19.76)*	40.24 *(14.92)*
Representational art (RA)	4.08 *(0.64)*	1160 *(380)*	37.59 *(15.26)*	42.47 *(16.54)*
Indeterminate art (IA)	4.35 *(0.76)*	1161 *(381)*	31.47 *(14.41)*	48.41 *(19.7)*
*Non-artists (NA)*				
Abstract art (AA)	3.32 *(1.11)*	931 *(329)*	58.47 *(24.89)*	28.11 *(22.62)*
Representational art (RA)	4.34 *(0.85)*	1056 *(344)*	31.53 *(20.93)*	51.16 *(21.66)*
Indeterminate art (IA)	4.37 *(0.97)*	1019 *(358)*	32.11 *(21.54)*	49.35 *(24.31)*

The mean rating of the level of affect was analyzed in a 2 (Group: NA, A) × 3 (Category of art: AA, RA, IA) ANOVA. This analysis revealed that the category of art had a significant effect on the rating of the mean level of affect, (*F*_(1.47, 49.95)_ = 14.49, *p* < 0.001, $\eta_{p}^{2}$ = 0.3). The interaction between the category of art and group indicated that the rating of affect was different between non-artists and artists, (*F*_(1.47, 49.95)_ = 5.69, *p* < 0.05 ${\text{η}}_{p}^{2}$ = 0.14), with differences evident in response to both AA and RA. Compared to artists, non-artists rated AA as having a lower mean level of affect and RA as having a higher mean level of affect. In order to investigate the interaction, an analysis of simple main effects revealed a significant effect of non-artists, (*F*_(1.19, 21.34)_ = 13.32, *p* < 0.01, ${\text{η}}_{p}^{2}$ = 0.43) on the mean rating of level of affect. Pairwise comparisons, using the Bonferroni procedure, indicated overall significant differences in the mean rating of the level of affect between AA and both RA and IA (both *p* < 0.01). There were no significant differences in the mean rating of affect between RA and IA. AA was rated as having a significantly lower mean level of affect than either RA or IA by the non-artists. There was also a significant effect for artists on the mean rating of the level of affect, (*F*_(1.82, 29.07)_ = 3.73, *p* < 0.05, ${\text{η}}_{p}^{2}$ = 0.19). However this difference was not reliable as the pairwise comparisons revealed that there were no significant differences in the mean rating of level of affect between different categories of art in artists.

In order to examine how the affective response impacted on the response time a 2 (Group: NA, A) × 3 (Category of art: AA, RA, IA) ANOVA was conducted. This analysis revealed that there was a significant main effect of the category of art on the time taken to respond, (*F*_(1.56, 53.08)_ = 18.61, *p* < 0.001, ${\text{η}}_{p}^{2}$ = 0.35) and that there was a significant interaction between the category of art and the two groups, (*F*_(1.56, 53.08)_ = 6.10, p *=* < 0.01, ${\text{η}}_{p}^{2}$ = 0.15). Pairwise comparisons found that the response times were significantly different between AA and both RA and IA (*p* < 0.001) but not between RA and IA. Exploring the interaction, further analysis of simple main effects found that the differences in the response times to the different categories of art were significant only in non-artists, (*F*_(1.37, 24.68)_ = 24.42, *p* < 0.001, ${\text{η}}_{p}^{2}$ = 0.58), representing a large effect. The Bonferroni procedure found that response times differed significantly between all three categories of art in this group. Thus, non-artists were significantly faster in rating the level of emotional affect of AA than either RA or IA, whereas there was no difference in the time taken to respond to any category of art by artists.

In order to compare extremes of affect between categories of art and between groups the mean frequency of pictures rated for low and high affect judgements was calculated separately. The mean frequency of pictures rated as having either low or high affect was analyzed in a 2 (Group; NA, A) × 3 (Category of art: AA, RA, IA) × 2 (Level of affect; low, high) ANOVA. There was a significant interaction between category of art and frequency of rating of level of affect, (*F*_(2, 68)_ = 14.61, *p* < 0.001, ${\text{η}}_{p}^{2}$ = 0.3). Pairwise comparisons, using the Bonferroni procedure, revealed significant differences in non-artists frequency of rating pictures having low or high affect. More AA was rated as having low than high affect (*p* < 0.05). Although more RA and IA pictures were rated as having high than low affect, only differences with regard to RA were near to significant (*p* = 0.054). Artists showed a very different pattern. The frequency of rating was significantly different only for IA (*p* < 0.05), with more IA rated as having high than low affect. The frequency of rating for AA showed almost no difference between high or low affect. There was also a significant interaction between the frequency of rating of level of affect, category of art, and group, (*F*_(2, 68)_ = 6.81, *p* < 0.01, ${\text{η}}_{p}^{2}$ = 0.17). Independent t tests revealed that non-artists significantly rated AA as having low affect more frequently than artists, (*t*_(34)_ = 2.52; *p* < 0.05), and there were no significant differences between groups in frequency of ratings of low affect for either RA or IA.

There were no significant differences in frequency of rating of high affect between the two groups.

## ERP Analysis

### Analysis of Non-Target Stimuli

*(See Supplementary Material for analysis of the pattern of ERP effects across group and non-target stimuli, RSS and FSS. For completeness Table 5a outlines all significant main effects and interactions)*.

### Analysis of Target Art Stimuli

An initial analysis was conducted to ensure that the number of trials missed by participants (i.e., when they did not respond, or when the data was rejected due to artifacts) was not significantly different between the groups for each category of art. No significant differences between the groups for any of the categories of art were found; AA, *t*_(34)_ = 1.92, *p* > 0.05, IA, *t*_(34)_ = 1.43, *p* > 0.05, RA, *t*_(34)_ = 0.63, *p* > 0.05.

The main focus of the present work was on the pattern of ERP effects across participant group (NA vs. A) and type of art (AA vs. RA and IA, and RA vs. IA). The mean amplitude and latency of each ERP component (P1, N1, P2, N2, P3, LPP) was analyzed in a 2 (Group: NA, A) × 3 (Stimuli: AA, RA, IA) × 3 (Hemisphere: left, midline, right (L, M, R)) × 4 (Location: frontal, central, parietal, occipital (F, C, P, O)) ANOVA.

*(For completeness Table 5b in Supplementary Material outlines all significant main effects and interactions)*.

**Figure 2:**
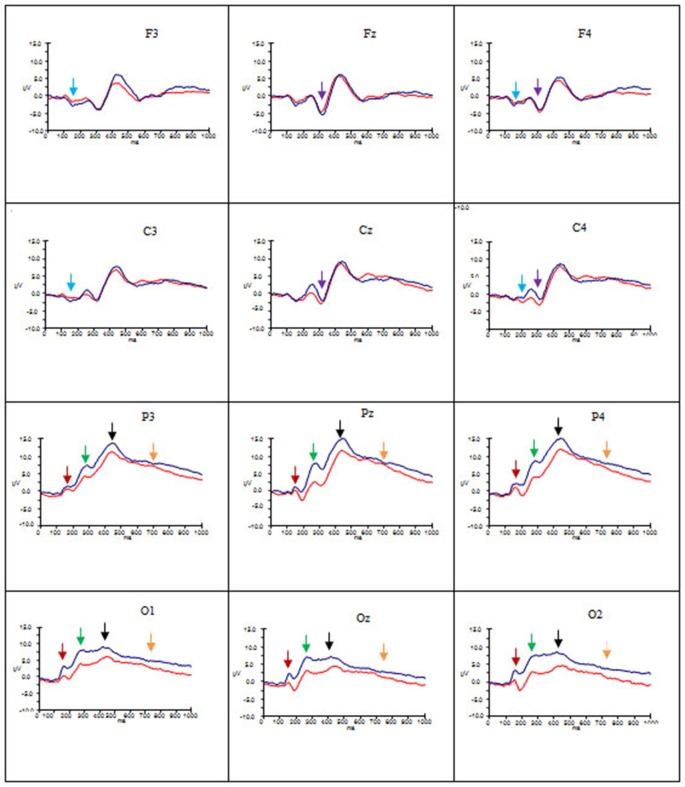
**Grand average ERP’s for AA for artists (blue) and non-artists (red).** Time 0–1000 ms, at selected frontal, central, parietal and occipital sites. Scale −10 ±15 μV. P1 component indicated by dark red arrow 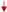; N1 component indicated by light blue arrow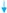; P2 component indicated by green arrow 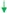; N2 component indicated by purple arrow 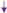; P3 component indicated by black arrow 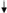; Late Positive Potential (LPP) indicated by gold arrow 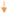.

**Figure 3:**
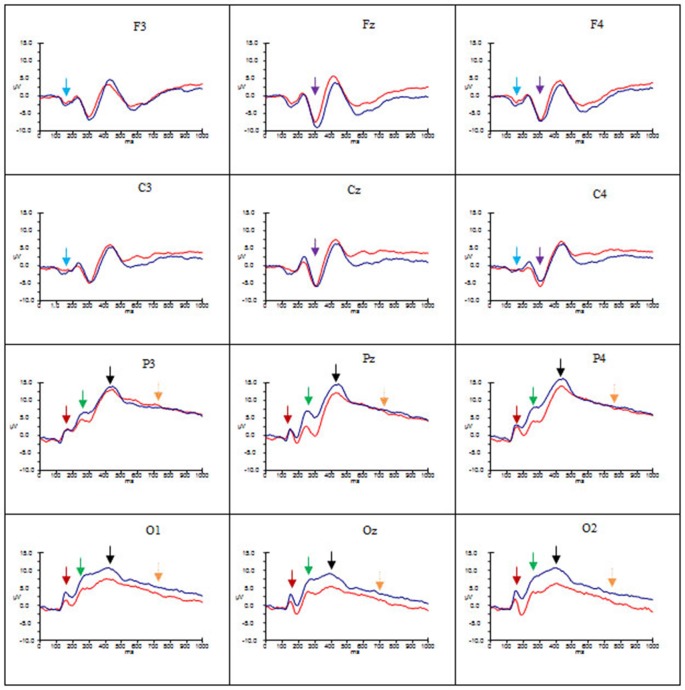
**Grand average ERP’s for RA for artists (blue) and non-artists (red).** Time 0–1000 ms, at selected frontal, central, parietal and occipital sites. Scale −10 *pm* 15 μV. P1 component indicated by dark red arrow 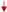; N1 component indicated by light blue arrow 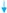; P2 component indicated by green arrow 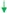; N2 component indicated by purple arrow 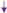; P3 component indicated by black arrow 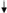; LPP indicated by gold arrow 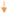.

**Figure 4:**
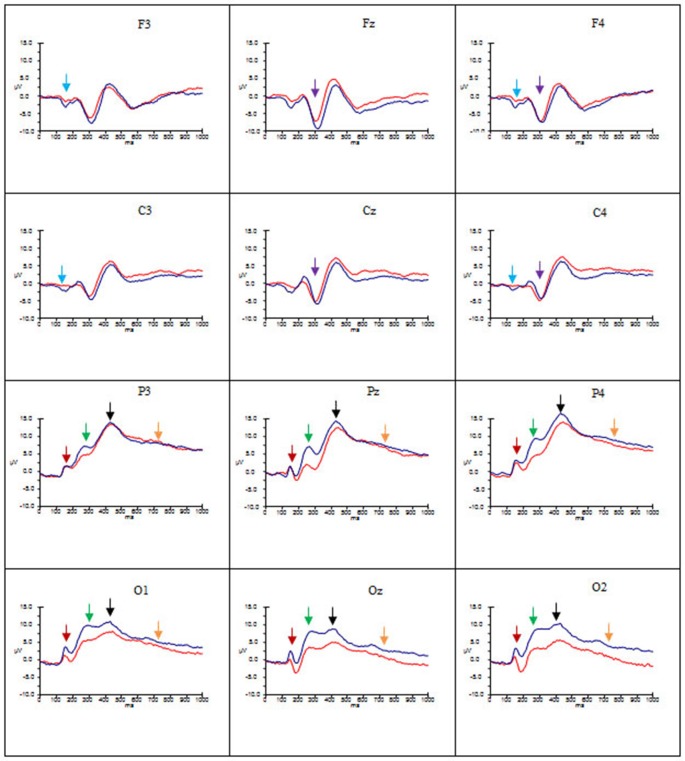
**Grand average ERP’s for IA for artists (blue) and non-artists (red).** Time 0–1000 ms, at selected frontal, central, parietal and occipital sites. Scale −10 ±15 μV. P1 component indicated by dark red arrow 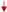; N1 component indicated by light blue arrow 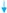; P2 component indicated by green arrow 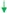; N2 component indicated by purple arrow 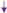; P3 component indicated by black arrow 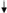; LPP indicated by gold arrow 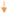.

Figures 2–4 show the grand average ERPs evoked in response to AA, RA, IA, respectively, for artists (blue) and non-artists (red) at selected frontal, central, parietal and occipital sites (F3, Fz, F4, C3, Cz, C4, P3, Pz, P4, O1, Oz, O2).

#### P1 Amplitude and Latency (100–180 ms)

Analysis of mean amplitude of P1 revealed a main effect of type of art (*F*_(2, 58)_ = 23.67, *p* < 0.001, ${\text{η}}_{p}^{2}$ = 0.45). Pairwise comparisons revealed more positivity for both RA and AA than for IA, (IA < AA, *p* < 0.001; IA < RA, *p* < 0.001). There was a significant interaction between type of art and location (*F*_(2.45, 71.15)_ = 15.04, *p* < 0.001, ${\text{η}}_{p}^{2}$ = 0.24) with occipital and parietal sites showing more positivity than frontal and central sites (P > F, *p* < 0.001; O > F, *p* < 0.001; P > C, *p* < 0.001; O > C, *p* < 0.001). There was also a significant interaction between group and location (*F*_(1.98, 57.28)_ = 4.71, *p* < 0.05, ${\text{η}}_{p}^{2}$ = 0.14), with artists showing larger amplitude at occipito-parietal locations for all types of art than non-artists. There was a significant three-way interaction between type of art, hemisphere and location (*F*_(3.88, 112.50)_ = 5.96, *p* < 0.05, ${\text{η}}_{p}^{2}$ = 0.08).

Analysis of the latency of the P1 revealed a main effect of type of art (*F*_(2, 58)_ = 27.61, *p* < 0.001, ${\text{η}}_{p}^{2}$ = 0.49) with increased latency for both AA and RA than for IA (AA > IA, *p* < 0.001; RA > IA, *p* < 0.001). There was a significant interaction between type of art and location (*F*_(6, 174)_ = 7.16, *p* < 0.001, ${\text{η}}_{p}^{2}$ = 0.20) with occipital, parietal and central locations showing a later peaking P1 than frontal (O > F, *p* < 0.005; P > F, *p* < 0.001; C > F, *p* < 0.005) and parietal locations peaking later than central (P > C, *p* < 0.005). There were three-way interactions between group and hemisphere and location, (*F*_(6, 174)_ = 2.45, *p* < 0.05, ${\text{η}}_{p}^{2}$ = 0.08), and between type of art and hemisphere and location, (*F*_(12, 348)_ = 2.42, *p* < 0.01, ${\text{η}}_{p}^{2}$ = 0.08).

Overall, these data point to group differences in both amplitude and latency of the P1 component. Artists demonstrated larger amplitude and latency of the P1 for all types of art at occipito-parietal locations than non-artists. Furthermore in terms of stimulus type the activation in artists is similar for all three types of art, whereas the activation in non-artists is dissimilar for all three. This pattern is evident from Figures 2–4 which indicate the P1 component at occipital and parietal locations with a red arrow.

#### N1 Amplitude and Latency (170–220 ms)

Analysis of the N1 mean amplitude revealed no significant main effects. However there were significant interactions between group and location, (*F*_(3, 87)_ = 4.58, *p* < 0.01, ${\text{η}}_{p}^{2}$ = 0.14), between type of art and hemisphere (*F*_(4, 116)_ = 3.38, *p* < 0.01, ${\text{η}}_{p}^{2}$ = 0.10), and a significant three-way interaction between type of art and hemisphere and location, (*F*_(12, 348)_ = 4.46, *p* < 0.001, ${\text{η}}_{p}^{2}$ = 0.13). Pairwise comparisons, revealed that artists showed more negativity than non-artists at frontal and central locations than at parietal (F > P, *p* < 0.05; C > P, *p* < 0.01), and more negativity in amplitude at left and right hemispheres than at mid line (L > M, *p* < 0.001, R > M, *p* < 0.001) for all types of art.

Analysis of the mean latency of the N1 revealed a significant main effect of type of art, (*F*_(1.34, 38.78)_ = 231.71, *p* < 0.001, ${\text{η}}_{p}^{2}$ = 0.89), with pairwise comparisons revealing increased latency for both AA and RA than for IA (AA > IA, *p* < 0.001; RA > IA, *p* < 0.001). There were significant interactions between both type of art and location, (*F*_(2.46, 71.39)_ = 7.19, *p* < 0.005, ${\text{η}}_{p}^{2}$ = 0.20), and type of art and hemisphere, (*F*_(3.11, 90.14)_ = 8.25, *p* < 0.001, ${\text{η}}_{p}^{2}$ = 0.22), with increased latency found at both mid line and right hemispheres compared to left (M > L, *p* < 0.01, R > L, *p* < 0.05). There was a significant three-way interaction between group and hemisphere and location (*F*_(2.85, 82.65)_ = 3.36, *p* < 0.05, ${\text{η}}_{p}^{2}$ = 0.10).

These data suggest that the N1 component was evident for artists only, with larger amplitude for this group for all types of art at frontal and central locations and left and right sites, whilst the latency was longer for AA and RA than for IA. This pattern is evident from Figures 2–4 which show the N1 at selected electrodes F3, F4, C3 and C4 with a blue arrow.

#### P2 Amplitude and Latency (190–300 ms)

Analysis of the amplitude of the P2 revealed a significant main effect of type of art, (*F*_(2, 58)_ = 23.10, *p* < 0.001, ${\text{η}}_{p}^{2}$ = 0.44), with mean amplitude being larger for both RA and AA than for IA (RA > IA, *p* < 0.001; AA > IA, *p* < 0.001). Mean amplitude did not differ between RA and AA. A significant main effect of group, (*F*_(1, 29)_ = 4.96, *p* < 0.05, ${\text{η}}_{p}^{2}$ = 0.15), demonstrated that mean amplitude was larger for artists than non-artists. There were significant interactions between group and type of art, (*F*_(1.97, 57.04)_ = 4.08, *p* < 0.05, ${\text{η}}_{p}^{2}$ = 0.12), group and location, (*F*_(1.91, 55.48)_ = 7.07, *p* < 0.005, ${\text{η}}_{p}^{2}$ = 0.20) and type of art and location, (*F*_(3.10, 89.75)_ = 17.98, *p* < 0.001, ${\text{η}}_{p}^{2}$ = 0.38). Finally, the three-way interaction between group and type of art and location, approached significance (*F*_(3.10, 89.75)_ = 2.65, *p* = 0.05, ${\text{η}}_{p}^{2}$ = 0.08). The group and type of art interaction shows that in both groups the mean P2 amplitude was larger for both AA and RA than for IA, however it was significantly greater for artists than non-artists for all three types of art. The type of art and location interaction revealed that whilst the P2 amplitude was larger at all locations for AA and RA than for IA, it was significantly larger at occipital and parietal locations compared to central and frontal locations (O > C, *p* < 0.001; O > F, *p* < 0.001; P > C, *p* < 0.001; P > F, *p* < 0.001) and at central compared to frontal locations (C > F, *p* < 0.005) for all types of art. The group and location interaction and inspection of the grand average charts (Figures 2–4) show that at all locations other than frontal the amplitude of the P2 was larger for artists than non-artists. Finally, the interaction between group and type of art and location approached significance.

Analysis of the mean latency of the P2 component revealed a significant main effect of type of art (*F*_(1.37, 39.70)_ = 178.98, *p* < 0.001, ${\text{η}}_{p}^{2}$ = 0.86), and a significant interaction between type of art and location (*F*_(2.58, 74.95)_ = 12.59, *p* < 0.001, ${\text{η}}_{p}^{2}$ = 0.30). The latency was greatest for AA, and smallest for IA (AA > RA, *p* < 0.05; AA > IA, *p* < 0.001; RA > IA, *p* < 0.001) at central and parietal locations compared to frontal, and at parietal compared to central locations (C > F, *p* < 0.05; P > F, *p* < 0.01; P > C, *p* < 0.05). There was also a three-way interaction between group and location and hemisphere (*F*_(2.88, 83.51)_ = 3.20, *p* < 0.05, ${\text{η}}_{p}^{2}$ = 0.10).

In summary, amplitude of the P2 was larger for AA and RA than for IA at parieto-occipital sites, in both groups, but for all three types of art amplitude of the P2 at occipital, parietal and central sites was greater for artists than non-artists. The P2 peaked earlier for IA and later for AA. This pattern is evident in Figures 2–4 showing the P2 component at parietal and occipital sites (P3, Pz, P4, O1, Oz, O2) indicated by a green arrow.

#### N2 Amplitude and Latency (275–350 ms)

Statistical analysis of the N2 component (275–350 ms) confirmed a significant main effect of type of art (*F*_(1.64, 47.64)_ = 178.98, *p* < 0.001, ${\text{η}}_{p}^{2}$ = 0.31). Also a significant interaction between both type of art and hemisphere (*F*_(3.13, 90.70)_ = 3.23, *p* < 0.05, ${\text{η}}_{p}^{2}$ = 0.10) and type of art and location (*F*_(2.04, 59.19)_ = 43.55, *p* < 0.001, ${\text{η}}_{p}^{2}$ = 0.60), and a significant three-way interaction between type of art, hemisphere and location (*F*_(5.53, 160.49)_ = 4.27, *p* < 0.005, ${\text{η}}_{p}^{2}$ = 0.13). Pairwise comparisons revealed significant differences between types of art, with larger negative amplitude for both RA and IA than for AA (RA > AA, *p* < 0.01; IA > AA, *p* < 0.001). Further analysis revealed that negative amplitude was larger in the right and mid line sites (M > L, *p* < 0.05; R > L, *p* < 0.05), than the left, and at frontal and central locations (F > C, *p* < 0.05; F > P, *p* < 0.001; F > O, *p* < 0.001; C > P, *p* < 0.001; C > O, *p* < 0.001) than at posterior locations. Finally, there was also a significant interaction between group and location (*F*_(1.78, 51.56)_ = 5.43, *p* < 0.01, ${\text{η}}_{p}^{2}$ = 0.16), with artists showing larger negative amplitude at central sites than non-artists.

With regard to latency of the N2 component there was also a significant main effect of type of art (*F*_(1.19, 34.44)_ = 596.27, *p* < 0.001, ${\text{η}}_{p}^{2}$ = 0.95), a significant interaction between type of art and hemisphere (*F*_(2.72, 78.88)_ = 6.01, *p* < 0.005, ${\text{η}}_{p}^{2}$ = 0.10), and significant three-way interactions between group, hemisphere and location (*F*_)2.71, 78.65)_ = 3.00, *p* < 0.05, ${\text{η}}_{p}^{2}$ = 0.09) and between type of art, hemisphere and location (*F*_)4.54, 131.68)_ = 3.31, *p* < 0.05, ${\text{η}}_{p}^{2}$ = 0.10). Latency was longer for both AA and RA than for IA (AA > IA, *p* < 0.001; RA > IA, *p* < 0.001), and at mid line and right sites than at the left (M > L, *p* < 0.005; R > L, *p* < 0.005). There was also a significant interaction between type of art, hemisphere, location and group (*F*_(4.54, 131.68)_ = 2.55, *p* < 0.05, ${\text{η}}_{p}^{2}$ = 0.08).

These findings indicate that there are significant differences both between types of art and group. Both groups demonstrate more negativity for RA and IA than for AA at frontal and central locations, and in mid line and right sites, whilst artists show larger amplitude than non-artists at these sites. The latency of this component, however, is longer for AA and RA than for IA. See Figures 2–4 showing the N2 at midline frontal and central sites with purple arrow.

#### P3 Amplitude and Latency (340–590 ms)

No significant main effects were revealed in the time window 340–590 ms. There was a significant interaction between group and location (*F*_(2.18, 63.25)_ = 3.64, *p* < 0.05, ${\text{η}}_{p}^{2}$ = 0.11), significant interactions between type of art and hemisphere (*F*_(2.60, 75.38)_ = 3.38, *p* < 0.05, ${\text{η}}_{p}^{2}$ = 0.10) and type of art and location (*F*_(2.66, 77.12)_ = 15.29, *p* < 0.001, ${\text{η}}_{p}^{2}$ = 0.35), and a three way interaction between group, type of art and location (*F*_(2.66, 77.12)_ = 4.92, *p* < 0.01, ${\text{η}}_{p}^{2}$ = 0.15). *Post hoc* analyses and inspection of the grand-average charts (Figures 2–4, P3 indicated by a black arrow) reveal that magnitude of the P3 was larger at parietal sites than at occipital, central or frontal sites (P > O, *p* < 0.001; P > C, *p* < 0.001; P > F, *p* < 0.001), and was larger at occipital sites than at frontal (O > F, *p* < 0.005) for artists than non-artists. There was enhanced positivity at parietal and occipital sites and at the right site for RA and IA compared to AA, in both groups, which was more pronounced for artists. However it can also be observed that the positivity in response to AA is more equally distributed across the three scalp sites than for the other two types of art and that when the differences between the two groups are inspected the *difference* in the amplitude is larger for artists in response to AA than non-artists, particularly at parietal sites.

With regard to latency of the P3 component there was a significant main effect of type of art (*F*_(1.5, 43.7)_ = 12.41, *p* < 0.001, ${\text{η}}_{p}^{2}$ = 0.30). Both AA and RA produced longer latencies in this time window than IA (AA > IA, *p* < 0.001; RA > IA, *p* < 0.005). There was also a three-way interaction between group, hemisphere and location (*F*_(2.85, 82.76)_ = 2.95, *p* < 0.05, ${\text{η}}_{p}^{2}$ = 0.09). Pairwise comparisons reveal longer latency in the mid line and right hemispheres than the left (M > L, *p* < 0.005; R > L, *p* < 0.01). Latency was longer at occipital sites than at parietal (O > P, *p* < 0.001; O > C, *p* < 0.05) and was longer at parietal and central sites than at frontal (P > F, *p* < 0.05; C > F, *p* < 0.005), in both groups. However, as before, these differences in latency are larger in artists than non-artists.

In summary, the effect of both type of art and group appear on both amplitude and latency of the P3 component. The amplitude was larger for all types of art at occipito-parietal sites for artists, however the distribution across the left, mid and right sites was more pronounced in response to AA for the artists than non-artists. With regard to the latency, the P3 latency was longer for both AA and RA than for IA in both groups, but overall, the latency was longer for all types of art and at occipital and parietal sites for artists than non-artists.

#### Late Positive Potential (LPP; 500–1000 ms)

**Figure 5:**
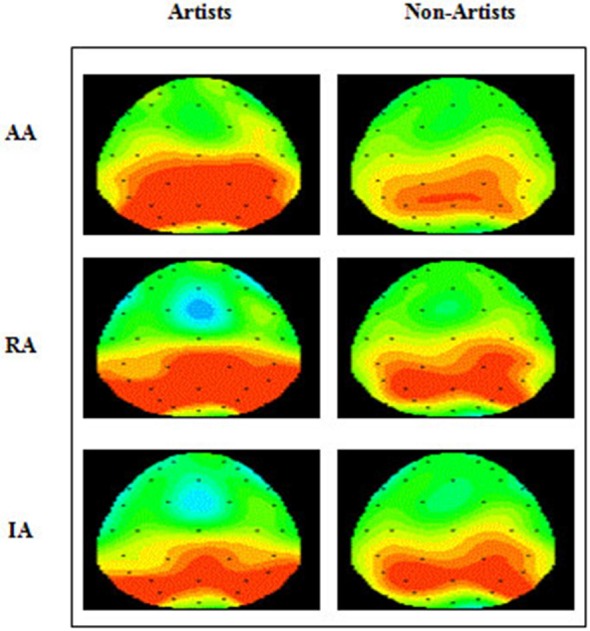
**Grand average topographic scalp maps showing ERP components for the late positive component, (LPP, at 1000 ms), for AA, RA and IA, for two groups, artists (A) and non-artist (NA) for 12 electrodes (F3, C3, P3, PZ, O1 OZ, O2, P4, C4, F4, FZ, CZ)**.

An ANOVA of the LPP also revealed no significant main effects, and only one significant interaction of interest, that between type of art and location (*F*_(2.45, 70.95)_ = 3.46, *p* < 0.05, ${\text{η}}_{p}^{2}$ = 0.11). *Post hoc* analysis revealed that amplitude was significantly larger in both left and right hemispheres than the mid line hemisphere (L > M, *p* < 0.01 ; R > M, *p* < 0.005), and that it was largest at parietal sites (P > O, *p* < 0.001; P > C, *p* < 0.001; P > F, *p* < 0.001), and larger at central and occipital sites than at frontal (C > F, *p* < 0.001; O > F, *p* < 0.005). Inspection of the topographic scalp maps (Figure 5) shows that at 1000 ms a large posterior effect can be observed, with larger amplitude at parietal sites for all types of art in both groups. In artists the amplitude in this location appears to be larger and more widespread than for non-artists, and larger for AA than for RA and IA, whilst for non-artists the amplitude is smaller for AA than for either of the others. Furthermore, inspection of the grand average charts (Figures 2–4, LPP indicated by a gold arrow) suggests that amplitude is generally larger at occipital sites for all categories of art for artists than for non-artists, is smaller in response to IA than to either AA or RA, and that the difference between the groups is most evident in response to IA.

To summarize, the amplitude of the LPP was largest at left and right parietal sites in response to all categories of art, in both groups, but artists demonstrated larger amplitude in response to AA at these sites. At occipital sites artists’ demonstrated larger amplitude than non-artists to all categories of art. Whilst it was larger for AA, the difference between the groups appears to be greater in response to IA.

## Discussion

### Main Results

The aim of this study was to investigate the electro-cortical correlates of expertise and the affect of modern art. Two groups of participants, artists and non-artists, viewed pictures of modern art in three categories, representational (RA), indeterminate (IA) and abstract (AA). They were asked to rate their immediate, emotional, visceral response whilst EEG was recorded. They also viewed two plain colored shapes, rare (RSS) and frequent (FSS) non-target stimuli, but were not required to make a response to these. RA allowed the investigation of visual art where clear semantic content may influence affect. AA has no recognisable content; emotional responses are simply to line, color and shape. Finally, IA represents a perceptual challenge, objects are not immediately, if at all, recognisable, like seeing faces or galloping horses in clouds.

The following main results were revealed. Frequency of rating the level of affect revealed that RA had greatest emotional effect on non-artists, whilst IA was rated as most affective by artists. Whilst AA was rated as evoking least affect in both groups, artists rated AA as having greater affect than non-artists. Art expertise was associated with larger ERP amplitudes in response to all categories of art. This positive expertise amplitude relationship was most evident in response to AA compared to either RA or IA. Where artists displayed an increase in mean amplitude in response to AA, non-artists displayed the converse, mean amplitude in this group decreased in response to AA compared to RA and IA. The N1, thought to index early visual processing of emotional stimuli, was evident only for artists. Furthermore, the N2, thought to index natural selective attention, was evident for RA and IA, but not AA, in both groups. The P3 and LPP, components most associated with affect and long term memory, were evident in both groups for all types of art, but the difference between groups was most evident in response to AA. Finally, lack of expertise appears to impact on the link between professed emotional affect and magnitude of the ERP. Non-artists demonstrated larger ERP amplitudes in response to RA and IA, art they rated as having greatest affect. This was not evident in artists.

#### Art and Affect

As predicted, non-artists rated affect of AA significantly lower, and significantly faster, than that for both RA and IA. On the other hand, there were no significant differences in either affect rating or time taken between the three categories of art by artists. The mean rating for IA was virtually the same in both groups. When we look at high and low affect ratings artists found almost equal numbers of AA to have high or low affect, and took almost the same time to make their decision as for the other categories of art, whilst the opposite was found in the non-artists, who, as expected, rated significantly more AA as having low affect than high (Pihko et al., 2011), and made their decision significantly faster than for the other two categories. This suggests that expertise encouraged more consideration and interest before a decision was made regarding affect. This supports findings of Vartanian and Goel (2004a) and Ishai et al. (2007) regarding aesthetic affect and preference, and also the proposal of Silvia (2009) that knowledge emotions such as confusion or surprise are involved in the appraisal of art. The affect ratings indicate a more equal emotional response from artists to the different categories of art, and as expected, a strong lack of emotional effect of AA on non-artists (Komar et al., 1997; Vartanian and Goel, 2004a; Cinzia and Vittorio, 2009; Pihko et al., 2011) suggesting that semantic content drives the emotional response to art, particularly in those with little art expertise.

With regard to RA and IA, non-artists rated virtually the same numbers as having high or low affect. But artists rated a significant number of IA as having high affect compared to low, compared to the other two categories. This suggests that visual indeterminacy is an important factor regarding affect (Ishai et al., 2007) and reflects the importance of visual ambiguity in art (Gregory, 1998). Non-artists rated RA as having greatest effect, suggesting that they responded to the real world content. Content rather than style has been found to be of central importance regarding the appreciation of RA (Augustin and Leder, 2006). It has previously been proposed that expertise enables greater discrimination of the style of AA and IA allowing for enhanced semantic processing, whilst art naive viewers depend more on content-related processing (Belke et al., 2006; Pihko et al., 2011) and refer to criteria such as personal feelings (Augustin and Leder, 2006). Leder et al. (2004) propose that the challenge of modern art requires additional stages of processing to result in an emotional response; the cognitive experience of modern art is more than simply an interesting perceptual process. The stimulation of successfully cognitively mastering the art results in the motivation to further expose oneself, increasing interest and expertise. Thus emotional responses to different categories of art appear to depend on art expertise (Augustin and Leder, 2006; Augustin et al., 2008; Cupchik et al., 2009; Pihko et al., 2011), particularly AA.

#### Art Expertise: Increased ERP Amplitude in Response to Art

In contrast with the findings of Pang et al. (2013), our results suggest that rather than expertise being associated with reduced ERP amplitude, it is the converse; greater expertise is associated with increased ERP amplitude. Whilst Pang et al. (2013) focussed on the P3 and LPP component in a passive task, here we found that not only did amplitude of the P3 increase in response to target stimuli, but overall, these data point to group differences in the amplitude of all six components analyzed, with artists demonstrating increased magnitude in response to all types of art than non-artists. Rather than expertise being associated with *reduced* neural responses, reflecting increased neural efficiency due to extensive practice, we suggest that art expertise is associated with *increased* neural responses reflecting greater sensitivity to emotional content, attention and memory resources. The increased attention artists allocate to stimuli is clearly demonstrated when we look at the analysis of the components P1, N1, P2, N2, P3 and LPC which reveal a number of interesting interactions.

Both the P1and N1are known to be sensitive to low-level features such as color, luminance and contrast (Anllo-Vento and Hillyard, 1996; Vogel and Luck, 2000; Bradley et al., 2007). None of these features were measured, controlled or manipulated in this study, as negligible changes can produce dramatic effects in art, particularly of luminance. Livingstone (2002) explains that the most primitive or necessary visual information is found in luminance variations, it determines depth, motion and spatial perception, and artists since the 13th century have employed luminance contrasts to enhance their art. Artists showed larger amplitude of the P1 than non-artists for all three categories of art, particularly at occipito-parietal sites. Furthermore in terms of stimulus type the activation in artists is similar for all three types of art, whereas the activation in non-artists is dissimilar, with almost no P1 evident in response to AA. This suggests that artists allocated more attention, may be more sensitive to differing levels of luminance and were more affected to all categories of art, than non-artists at this very early time point (Carretié et al., 2004; Delplanque et al., 2004). The P1 has been reported in response to unpleasant pictures at occipital sites (Smith et al., 2003; Carretié et al., 2004; Delplanque et al., 2004). Here, whilst reasonable to suggest that artists would find visual art generally more affective than non-artists, it is unlikely that they would find it more unpleasant. On this occasion the P1 appears to be in response to emotionally affective stimuli, pleasant or unpleasant. The lack of evidence of a P1 in response to AA in non-artists, art rated as least affective, supports this.

The N1 was evident only for artists, for all categories of art. The visual N1, indexed to the allocation of attentional resources, selection and discrimination (Anllo-Vento and Hillyard, 1996; Vogel and Luck, 2000), and to early visual processing of emotional stimuli (Keil et al., 2002; Carretié et al., 2007; Foti et al., 2009; Weinberg and Hajcak, 2010), has also been associated with expertise (Bigman and Pratt, 2004, as cited in Pratt, 2012). A later N1 (about 170 ms) component has been reported in response to dogs and birds in experts (Tanaka and Curran, 2001; Busey and Vanderkolk, 2005), and linked to face processing and recognition (Rossion and Jacques, 2008). Here, we can conclude the artists allocated greater attentional resources, were more emotionally affected by all three categories of art, and were more expert at examining stimuli for recognisable objects or content, than non-artists.

Whilst the P2 and N2 components were evident for both groups, again amplitude was larger for artists than non-artists, in response to all art. The difference was most evident in response to AA at occipito-parietal sites for the P2, and at central sites for the N2 for RA and IA. This suggests that whilst both groups were responsive to emotional evaluation and affect of the artworks (Luck and Hillyard, 1994; Carretié et al., 2001, 2004; Delplanque et al., 2004; Olofsson and Polich, 2007), the effect was greater for artists. An enhanced P2 has been found in non-affective research in response to target stimuli, particularly infrequent targets (Luck and Hillyard, 1994), but early studies examining ERP responses to affective line drawings indicate that the magnitude of the P2 is sensitive to affective evaluation (Begleiter et al., 1979). Our findings support this. An increased P2 in response to AA in artists suggests they found AA more affective than non-artists, and their behavioral response reflected this. The N2 appears sensitive to the salience of the stimuli (Codispoti et al., 2006; Luck, 2012), emotion (Schupp et al., 2003a,b, 2004, 2006; de Tommaso et al., 2008; Olofsson and Polich, 2007; Foti et al., 2009; Weinberg and Hajcak, 2010) and has been observed when subjects search stimuli stored in visual working memory (Dell’acqua et al., 2010). Hajcak et al. (2012) report an early positive negativity (EPN) in the time range of the N2 generally observed following emotional content, related to increased selective attention. Accumulating evidence suggests that it may be particularly sensitive to pleasant rather than to unpleasant or neutral content. The increase in magnitude here suggests that it is in response to emotional valence. Artists were more engaged and affected than non-artists, particularly in response to IA and RA, which they also reported to be the most affective categories of art.

The findings of two later ERP components of interest, the P3 and LPP may reflect the role of knowledge in long-term memory and appreciation of art (Leder et al., 2004). The focus here is on attention and working memory operations during cognitive task performance, particularly those sensitive to emotional processing of visual stimuli. We observed an increase in magnitude of the P3 in response to evaluation of affect for all art, at occipito-parietal sites. The presence of this component, thought to be heavily dependent on attention (Hajcak et al., 2012), linked to context updating in working memory (Donchin and Coles, 1988) knowledge in long-term memory (Leder et al., 2004) and categorization (Luck, 2012), suggests that participants in both groups were attentively processing all three categories of art. Latency was longer for both AA and RA than for IA in both groups. Again, the effect was larger for artists than for non-artists, and more pronounced for AA. This suggests that the evaluation of affect of art appears to induce a higher level of attention and arousal in artists than in non-artists (Duncan-Johnson and Donchin, 1977; Polich, 2007), with the effect being more evident for AA, art with no semantic content. The P3 has previously been reported to be sensitive to beauty and aesthetic discrimination of geometric shapes (de Tommaso et al., 2008). The increased positivity in artists here reflects their higher affective rating of art, with the difference between the groups most evident in response to AA.

Although the data point to only one significant interaction with regard to the LPP, the topographic maps and the almost significant interactions point to a larger centro-parietal LPP for all art in artists than non-artists, with the difference most evident in response to AA. This long lasting increased ERP positivity in response to arousing pictures has often been observed (Mini et al., 1996; Cuthbert et al., 2000; Schupp et al., 2000; Keil et al., 2002; Foti et al., 2009), and again is associated with top-down processing influences, evaluation (Hajcak et al., 2006; Moser et al., 2006; Krompinger et al., 2008) and subjective emotional experience (Luck, 2012).

These results indicate that magnitude of the P3 and LPP increased in response to all art during affective evaluation, with the effect more pronounced in artists. This appears to contradict the findings of Pang et al. (2013). However, their study asked participants to simply view the paintings and stimuli, whereas on this occasion participants were required to make an affective evaluation, known to have a positive effect on the magnitude of these components (Cuthbert et al., 2000; Schupp et al., 2004; Weinberg and Hajcak, 2010). Nevertheless, our findings suggests that not only does expertise effect the evaluation of art, engagement is also required; simply looking at art may not be enough to experience its affect. Appreciating art appears to reflect the role of knowledge in long-term memory and involves top down processing.

#### Expertise and Abstract Art

The positive expertise amplitude relationship was most evident in response to AA. Where artists displayed an increase in mean amplitude of the ERP component in response to AA compared to RA and IA, non-artists displayed the converse, the mean amplitude in this group decreased. This is particularly evident for N1, P2, P3 and LPP. This difference suggests that the early attention of artists was engaged by AA (Carretié et al., 2004; Delplanque et al., 2004), they allocated greater attentional resources (Hillyard and Anllo-Vento, 1998; Luck and Kappenman, 2012), were more adept at engaging higher order visual processing (Luck and Hillyard, 1994; Carretié et al., 2001, 2004), experienced greater emotional arousal (Duncan-Johnson and Donchin, 1977; Polich, 2007; de Tommaso et al., 2008) and that their expertise influenced top-down processing, specifically in response to AA (Hajcak et al., 2006; Moser et al., 2006). The opposite was true in non-artists. This is both in line with their subjective arousal ratings and supports previous research (Cuthbert et al., 2000; Schupp et al., 2004; Vartanian and Goel, 2004a,b; Weinberg and Hajcak, 2010; Pihko et al., 2011). It also suggests that not only knowledge and experience, but great effort is required to appreciate AA (Augustin and Leder, 2006; Belke et al., 2006), whilst for those with little knowledge and experience semantic meaning is necessary (Vartanian and Goel, 2004a; Belke et al., 2006; Cinzia and Vittorio, 2009; Vessel and Rubin, 2010).

#### The N2; not Evident for AA

As we expected, the N2 amplitude was influenced by category of art. In both groups the N2 was evident in response to RA and IA, with larger amplitude at central sites, but not evident in response to AA. The N2 appears to index natural selective attention (Dolcos and Cabeza, 2002; Schupp et al., 2004; Codispoti et al., 2006) and is responsive to emotional stimuli (Olofsson and Polich, 2007). Here the N2 may have been responsive to valence, with larger amplitude observed in response to RA and IA, art rated as having most affect by both groups. Alternatively, this effect may simply be in response to the perceived semantic content of the art-works, supporting the proposal that familiar content, memory and mental imagery are all important regarding the appreciation of art (Augustin and Leder, 2006; Fairhall and Ishai, 2008). As AA contained no recognisable objects to stimulate natural selective attention there was nothing to run away from, nothing to eat, nothing to mate with, so no N2 component.

#### Increased ERP Amplitude for Affective Art

Previous studies using visual art as stimuli to explore aesthetic judgement and preference (Vartanian and Goel, 2004a,b; Di Dio et al., 2007; Kirk, 2008; Kirk et al., 2009a,b), aesthetic perception and beauty (Kawabata and Zeki, 2004; de Tommaso et al., 2008; Ishizu and Zeki, 2011) and the appeal of visual art (Lacey et al., 2011), have reported that looking at visual art (not necessarily beautiful) activates the reward-related areas of the brain. Lacey et al. (2011) propose that the appeal of visual art is based on its artistic status alone, not necessarily its aesthetic value, its beauty or its valence. These results support this notion, with a proviso. Looking at visual art with semantic content is rewarding, even if you have little or no knowledge (Vartanian and Goel, 2004a; Belke et al., 2006; Di Dio and Gallese, 2009; Vessel and Rubin, 2010), but not AA. However knowledge enhances that experience to include AA. Whilst both expertise and great effort are required to be able to sustain interest and to appreciate AA (Augustin and Leder, 2006; Belke et al., 2006), simply effort appears to be required to experience the emotions aroused by IA and RA.

## Conclusion

In response to affective art the amplitude of both endogenous and exogenous ERP components increased in both groups. Artists demonstrated larger ERP magnitude in response to all categories of art than non-artists, although they did not rate all art as having more affect. Their increased stimulation is evidenced by enhanced perceptual and emotional responses, supporting our hypotheses. Rather than expertise having a negative correlation with amplitude (Pang et al., 2013), expertise appears to have a positive effect, with increased magnitude of P3 and LPP at centro-parietal sites indexing greater emotional arousal. This expertise effect is particularly evident in response to AA. Due to artists knowledge and expectations reward-related areas of the brain were activated (Kirk et al., 2009a,b; Lacey et al., 2011), resulting in increased attentional demand (Lengger et al., 2007; Kirk, 2008) despite lack of semantic content. These results support Leder et al. (2004), regarding challenges, cognitive mastering and evaluation of modern art. Whereas attention of non-artists faded quickly, experts remained engaged, whether they found art affective or not.

We proposed that beauty, or aesthetic pleasantness is not the most important aspect of appreciating modern art, but that positive or negative affect is (Silvia, 2009). Our results support this hypothesis, with increased magnitude of ERP components associated with emotional response evident for all art in artists, and that rated as affective in non-artists. Future research studying judgement of modern art should consider a range of emotions. To concentrate on beauty or pleasantness may degrade the power of art.

A major limitation regarding this and previous studies is that, in fact, they are not using visual art as stimuli, but reproductions. The original size of the 300 artworks reproduced here ranged from 27 cm × 20 cm to over 3.5 m × 2.5 m but were displayed within 1931 × 1931 mm format on a computer monitor. Reproducing art immediately diminishes its impact, rendering it to the status of interesting stimuli, no longer art. The whole intent of the artwork is compromised as soon as it is reproduced, no matter how well. Original paintings viewed in a gallery have previously been rated as more pleasant or interesting than their reproduced counterparts (Locher et al., 2001). Despite logistical and paradigm problems research should endeavor to explore neurophysiological reactions to visual art using originals. Research into viewing habits of gallery visitors suggests that the average time spent contemplating art in galleries is 30 s (Locher et al., 2007). Although differences between schools of art have been identified in 1 ms (Bachmann and Vipper, 1983, as cited in Augustin et al., 2008), and here we demonstrated differences in visual and visceral responses in presentation times of less than 1500 ms, perhaps these differences are not specific to art, but are simply in response to visual stimuli. Most art is created to be contemplated, to be thought provoking, and to engage. In order to ensure responses are to art longer presentation times could be employed, in art galleries. Finally, the impact of expertise could be further explored. Does expertise impact on visual and affective processes more generally? Do art experts see faster, differently, more?

We found that looking at art is interesting and rewarding, particularly for artists, and does not depend upon aesthetic preference. We report increased amplitude of ERPs sensitive to emotional content in response to modern art in two groups, artists and non-artists, with an enhanced effect in artists. Both groups report that IA and RA had greatest affect, which is supported by the ERP magnitude. However, differences between groups are most evident in response to AA, suggesting that expertise is important regarding appreciation of AA.

## Conflict of Interest Statement

The authors declare that the research was conducted in the absence of any commercial or financial relationships that could be construed as a potential conflict of interest.
