# Supplementary material for: Art expertise modulates the emotional response to modern art, especially abstract: an ERP investigation
Source: Front Hum Neurosci. 2015 Sep 30;9:525. doi: 10.3389/fnhum.2015.00525 (PMC4876367; doi:10.3389/fnhum.2015.00525)
Supplement: Supplementary file 1 [file DataSheet_1.DOC]

**Supplementary materials**

1. Full catalogue of artworks used as stimuli

| **Artist** | **Title** | **Medium** | **Collection** |
| --- | --- | --- | --- |
| Joseph Albers | Study for Homage to the Square: Beaming 1963 | Oil on plastic and board | [www.tate.org.uk](http://www.tate.org.uk/) |
| Josef Albers | Circle 1933 | Woodcut on paper | [www.tate.org.uk](http://www.tate.org.uk/) |
| [Anselm Kiefer](http://www.tate.org.uk/servlet/ArtistWorks?cgroupid=999999961&artistid=1406&page=1) | Let a Thousand Flowers Bloom  2000 | Mixed media | [www.tate.org.uk](http://www.tate.org.uk/) |
| Karel Appel | Amorous Dance  1955 | oil on canvas | [www.tate.org.uk](http://www.tate.org.uk/) |
| Karel Appel | Untitled  1960 | Lithograph on paper | [www.tate.org.uk](http://www.tate.org.uk/) |
| Karel Appel | Hip, Hip, Hoorah!  1949 | Oil on canvas | [www.tate.org.uk](http://www.tate.org.uk/) |
| Eugene Atget | Bords de la Marne 1903 | Gelatin silver printing-out-paper print, | [www.moma.org](http://www.tate.org.uk/) |
| Frank Auerbach | Bacchus and Ariadne  1971 | Oil on board | [www.tate.org.uk](http://www.tate.org.uk/) |
| Frank Auerbach | Small Head of E.O.W.  1957-8 | Oil on board | [www.tate.org.uk](http://www.tate.org.uk/) |
| Frank Auerbach | The Sitting Room  1964 | Oil on board | [www.tate.org.uk](http://www.tate.org.uk/) |
| Francis Bacon | Portrait of Isabel Rawsthorne  1966 | Oil on canvas | [www.moma.org](http://www.moma.org/) |
| Francis Bacon | Study of a Dog  1952 | Oil on canvas | [www.tate.org.uk](http://www.tate.org.uk/) |
| Francis Bacon | Figure in a Landscape  1945 | Oil on canvas | [www.tate.org.uk](http://www.tate.org.uk/) |
| Francis Bacon | Reclining Woman 1961 | Oil on canvas | [www.tate.org.uk](http://www.tate.org.uk/) |
| Balthus | Sleeping Girl  1943 | Oil on board | [www.tate.org.uk](http://www.tate.org.uk/) |
| Balthus | Still Life with a Figure  1940 | Oil on paper mounted on wood panel | [www.tate.org.uk](http://www.tate.org.uk/) |
| Matthew Barney | Drawing Restraint 9: Shimenawa 2005 | Chromogenic color print in self-lubricating plastic frame, | [www.moma.org](http://www.tate.org.uk/) |
| Georg Baselitz | Adieu 1982 | Oil on canvas | [www.tate.org.uk](http://www.tate.org.uk/) |
| Jean-Michel Basquiat | Untitled 1981 | Oilstick on paper | [www.moma.org](http://www.tate.org.uk/) |
| Jean-Michel Basquiat | Untitled, 1985 | Cut-and-pasted paper and oilstick on paper, | [www.moma.org](http://www.tate.org.uk/) |
| Georg Baselitz | Rebel 1965 | Oil on canvas | [www.tate.org.uk](http://www.tate.org.uk/) |
| Bernd Becha and Hilla Becha | Pitheads  1974 | Photograph on board | [www.tate.org.uk](http://www.tate.org.uk/) |
| Thomas Hart Benton | Homestead 1934 | Tempera and oil on composition board | [www.moma.org](http://www.tate.org.uk/) |
| Joseph Beuys | Acer platanoides  1945 | Leaf on paper | [www.tate.org.uk](http://www.tate.org.uk/) |
| Joseph Beuys | Sun and Pylon  1946 | Pencil, watercolour and chloride on paper | [www.tate.org.uk](http://www.tate.org.uk/) |
| Umberto Boccioni | The City Rises, 1910 | Oil on canvas | [www.moma.org](http://www.tate.org.uk/) |
| [Alighiero e Boetti](http://www.moma.org/collection/artist.php?artist_id=630) | Map of the World 1989 | Embroidery on fabric | [www.moma.org](http://www.tate.org.uk/) |
| David Bomberg | In the Hold  circa 1913-4 | Oil on canvas | [www.tate.org.uk](http://www.tate.org.uk/) |
| David Bomberg | Bathing Scene  circa 1912-13 | Oil on wood | [www.tate.org.uk](http://www.tate.org.uk/) |
| Pierre Bonnard | Basket of Fruit Reflected in the Mirror, 1944-46 | Oil on canvas | [www.moma.org](http://www.tate.org.uk/) |
| Pierre Bonnard | The Bowl of Milk c 1919 | Oil on canvas | [www.tate.org.uk](http://www.tate.org.uk/) |
| Pierre Bonnard | Pont de la Concorde 1913/15 | Oil on canvas | [www.tate.org.uk](http://www.tate.org.uk/) |
| Pierre Bonnard | The Window 1925 | Oil on canvas | [www.tate.org.uk](http://www.tate.org.uk/) |
| Pierre Bonnard | Coffee 1915 | Oil on canvas | [www.tate.org.uk](http://www.tate.org.uk/) |
| Louise Bourgeois | Untitled 1989-91 | Drypoint etching on paper | [www.tate.org.uk](http://www.tate.org.uk/) |
| Constantin Brancusi | Untitled Interior of Studio 1922 | Gelatin silver print | [www.moma.org](http://www.tate.org.uk/) |
| Constantin Brancusi | Viewof the Artist's Studio 1918 | Gouache and pencil on board | [www.moma.org](http://www.tate.org.uk/) |
| Bill Brandt | Evening in Kew Gardens c.1935 | Gelatin silver print, | [www.moma.org](http://www.tate.org.uk/) |
| Georges Braque | Glass on a Table 1909/10 | Oil on canvas | [www.tate.org.uk](http://www.tate.org.uk/) |
| Henri Cartier-Bresson | Arsila, Spanish Morocco 1933 | Gelatin silver print, printed 1947 | [www.moma.org](http://www.tate.org.uk/) |
| Gunter Brus& Arnolf Rainer | Charm - Flower - Ring  1984 | Drawing on paper | [www.tate.org.uk](http://www.tate.org.uk/) |
| Arnulf Rainer & Gunter Brus | from Deepening with Clouding Over (P77235-P77239; complete) [no title] 1985-6 | Intaglio print on paper | [www.tate.org.uk](http://www.tate.org.uk/) |
| Chris Burden | Creatures Beyond Fathom of Science 1979 | Cut-and-pasted printed paper, gelatin silver print, snakeskin, and colored pencil on paperboard, | [www.moma.org](http://www.tate.org.uk/) |
| Anthony Caro | Figure 1956 | Monotype on paper | [www.tate.org.uk](http://www.tate.org.uk/) |
| Paul Cezanne | The Avenue at the Jas de Bouffan  circa 1874-5 | Oil on canvas | [www.tate.org.uk](http://www.tate.org.uk/) |
| Paul Cezanne | Boy in a red vest 1888-90 | Oil on canvas | [www.moma.org](http://www.tate.org.uk/) |
| Paul Cezanne | The Grounds of the Château Noir  circa 1900-6 | Oil on canvas | [www.tate.org.uk](http://www.tate.org.uk/) |
| Marc Chagall | I and the Village 1911 | Oil on canvas | [www.moma.org](http://www.tate.org.uk/) |
| Marc Chagall | Bouquet with Flying Lovers  circa 1934-47 | Oil on canvas | [www.tate.org.uk](http://www.tate.org.uk/) |
| Jake and Dino Chapman | from Exquisite Corpse (P78455-P78474; complete) Exquisite Corpse  2000 | Etching on paper | [www.tate.org.uk](http://www.tate.org.uk/) |
| Chuck Close | Self-Portrait/Pulp/Pochoir 2000 | Paper pulp and pochoir, composition and sheet: | [www.moma.org](http://www.tate.org.uk/) |
| Joseph Cornell | Untitled (Bird Box) about 1948 | Mixed-media assemblage in glass-fronted wooden box with electric light | [www.nationalgalleries.org](http://www.tate.org.uk/) |
| John Currin | The Wizard circa 1994 | Oil on canvas | [www.tate.org.uk](http://www.tate.org.uk/) |
| John Currin | Thanksgiving  2003 | Oil on canvas | www.tate.org.uk |
| Salvador Dali | Autumnal Cannibalism 1936 | Oil on canvas | [www.tate.org.uk](http://www.tate.org.uk/) |
| Salvador Dali | Mountain Lake 1938 | Oil on canvas | [www.tate.org.uk](http://www.tate.org.uk/) |
| Salvador Dali | Metamorphosos of Narcissus 1937 | Oil on canvas | [www.tate.org.uk](http://www.tate.org.uk/) |
| Willem de Kooning | Minnie Mouse 1971 | Lithograph | [www.moma.org](http://www.tate.org.uk/) |
| Giorgio De Chirico | The Painter's Family 1926 | Oil on canvas | [www.tate.org.uk](http://www.tate.org.uk/) |
| Giorgio De Chirico | The Uncertainty of the Poet, 1913 | Oil on canvas | [www.tate.org.uk](http://www.tate.org.uk/) |
| Robert Delauney | Study for `The City'  1909-10 | Oil on canvas | [www.tate.org.uk](http://www.tate.org.uk/) |
| Robert Delauney | Windows Open Simultaneously (First Part, Third Motif)  1912 | Oil on canvas | [www.tate.org.uk](http://www.tate.org.uk/) |
| Robert Delauney | Endless Rhythm  1934 | Oil on canvas | [www.tate.org.uk](http://www.tate.org.uk/) |
| Charles Demuth | Eggplant and Tomatoes 1926 | Watercolour on paper | [www.moma.org](http://www.tate.org.uk/) |
| Richard Diebenkorn | from Five Aquatints with Drypoint (P07644; incomplete) #4 1978 | Etching, aquatint and drypoint on paper | [www.tate.org.uk](http://www.tate.org.uk/) |
| Otto Dix | The Nun 1914 | Oil on cardboard | [www.moma.org](http://www.tate.org.uk/) |
| Otto Dix | Cardplayers | Drypoint on paper (4/11) | [www.nationalgalleries.org](http://www.moma.org/) |
| Peter Doig | Ski Jacket 1994 | Oil on canvas | [www.tate.org.uk](http://www.moma.org/) |
| Peter Doig | rom Cubitt Print Box (P78388-P78407; complete) Echo Lake 2000 | Etching and aquatint on paper | [www.tate.org.uk](http://www.moma.org/) |
| Jean Dubuffet | The Busy Life 1953 | Oil on canvas | www.tate.org.uk |
| Jean Dubuffet | Carrot Nose, 1962 | Lithograph | www.moma.org |
| Jean Dubuffet | Monsieur Plume with Creases in his Trousers (Portrait of Henri Michaux)  1947 | Oil and grit on canvas | www.tate.org.uk |
| Jean Dubuffet | The Tree of Fluids  1950 | Oil on canvas | [www.tate.org.uk](http://www.tate.org.uk/) |
| Jean Dubuffet | Large Black Landscape 1946 | Oil on board | [www.tate.org.uk](http://www.tate.org.uk/) |
| Marlene Dumas | Lucy 2004 | Oil on canvas | [www.tate.org.uk](http://www.tate.org.uk/) |
| Marlene Dumas | Magdalena 1  1996 | Ink on paper | [www.tate.org.uk](http://www.tate.org.uk/) |
| Tracy Emin | from Other Men's Flowers (P11422-P11436; complete) (no title) 1994 | Lithography on paper | [www.tate.org.uk](http://www.tate.org.uk/) |
| James Ensor | Effect of Light 1935 | Oil on canvas | [www.tate.org.uk](http://www.tate.org.uk/) |
| Max Ernst | Forest and Dove 1927 | Oil on canvas | [www.tate.org.uk](http://www.tate.org.uk/) |
| Max Ernst | Men Shall Know Nothing of This  1923 | Oil on canvas | [www.tate.org.uk](http://www.tate.org.uk/) |
| Walker Evans | City Lunch Counter, New York 1929 | Gelatin silver print, | [www.moma.org](http://www.tate.org.uk/) |
| Lyonel Feininger | Gelmeroda III 1913 | Oil on canvas | [www.nationalgalleries.org](http://www.tate.org.uk/) |
| Fischli & Weiss | from Fotografías (P20330-P20333; incomplete) Untitled 2005 | Photograph on paper | [www.tate.org.uk](http://www.tate.org.uk/) |
| Dan Flavin | Untitled 1973 | Crayon and ink on graph paper on paper, | [www.moma.org](http://www.tate.org.uk/) |
| Lucio Fontana | Spatial Concept 1958 | Pastel and canvas collage | [www.tate.org.uk](http://www.tate.org.uk/) |
| Lucien Freud | Girl with a White Dog 1950-51 | Oil on canvas | [www.tate.org.uk](http://www.tate.org.uk/) |
| Lucien Freud | Francis Bacon 1952 | Oil on canvas | [www.tate.org.uk](http://www.tate.org.uk/) |
| Gilbert & George | Existers  1984 | Mixed media | [www.tate.org.uk](http://www.tate.org.uk/) |
| Gilbert & George | Faith Drop  1991 | Mixed media | [www.tate.org.uk](http://www.tate.org.uk/) |
| Henri Gaudia-Breska | Leopard I  circa 1912-13 | Drawing on paper | [www.tate.org.uk](http://www.tate.org.uk/) |
| Paul Gauguin | Auti Te Pape (Women at the River) from Noa Noa (Fragrance) | Woodcut on paper | [www.moma.org](http://www.tate.org.uk/) |
| Isa Genzken | Fischcollage (#7) 2001 | Cut-and-pasted printed paper on paper | [www.moma.org](http://www.tate.org.uk/) |
| Alberto Giacometti | The Studio, 1955 | Lithograph | [www.moma.org](http://www.moma.org/) |
| Robert Gober | Untitled  2000 | Lithograph on paper | [www.tate.org.uk](http://www.tate.org.uk/) |
| Nan Goldin | Vivienne in the green dress, NYC  1980 | Photograph on paper | [www.tate.org.uk](http://www.tate.org.uk/) |
| Juan Gris | The Sunblind 1914 | Gouache, collage, chalk and charcoal on canvas | [www.tate.org.uk](http://www.tate.org.uk/) |
| Juan Gris | Overlooking the Bay  1921 | Oil on canvas | [www.tate.org.uk](http://www.tate.org.uk/) |
| Georg Grosz | Suicide  1916 | Oil on canvas | [www.tate.org.uk](http://www.tate.org.uk/) |
| Georg Grosz | A Married Couple  1930 | Watercolour on paper | [www.tate.org.uk](http://www.tate.org.uk/) |
| Andreas Gursky | Bahrain I  2005 | Photograph on paper | [www.tate.org.uk](http://www.tate.org.uk/) |
| Philip Guston | Hat 1976 | Oil on canvas | [www.tate.org.uk](http://www.tate.org.uk/) |
| Philip Guston | The Return 1956-8 | Oil on canvas | [www.tate.org.uk](http://www.tate.org.uk/) |
| Richard Hamilton | Chromatic spiral  1950 | Oil on wood | [www.tate.org.uk](http://www.tate.org.uk/) |
| Barbara Hepworth | Kestor Rock, Gleaming Stone  1973 | Lithograph on paper | [www.tate.org.uk](http://www.tate.org.uk/) |
| Barbara Hepworth | Genesis  1969 | Lithograph on paper | [www.tate.org.uk](http://www.tate.org.uk/) |
| Barbara Hepworth | Family Group - Earth Red and Yellow  1953 | Oil and drawing on board | [www.tate.org.uk](http://www.tate.org.uk/) |
| Barbara Hepworth | Two Forms (White and Yellow)  1955 | Oil and drawing on board | [www.tate.org.uk](http://www.tate.org.uk/) |
| Eva Hesse | Untitled 1967 | Drawing on paper | [www.tate.org.uk](http://www.tate.org.uk/) |
| Damien Hirst | Round from In a Spin, the Action of the World on Things, Volume 1, 2002 | One from a portfolio of twenty-three etching, aquatint, and drypoints, | [www.moma.org](http://www.tate.org.uk/) |
| Damien Hirst | from London (P77924-P77934; complete) Untitled 1992 | Screenprint on paper | [www.tate.org.uk](http://www.tate.org.uk/) |
| Damien Hirst | from In a Spin, the Action of the World on Things I (P13034-P13056; complete) Global a Go-Go - for Joe 2002 | Colour etching | [www.tate.org.uk](http://www.tate.org.uk/) |
| David Hockney | Rocky Mountains and Tired Indians 1965 | Acrylic on canvas | [www.nationalgalleries.org](http://www.tate.org.uk/) |
| Howard Hodgkin | Come into the Garden, Maude 2000-3 | Oil on wood | [www.tate.org.uk](http://www.tate.org.uk/) |
| Howard Hodgkin | Dinner at West Hill  1964-6 | Oil on canvas | [www.tate.org.uk](http://www.tate.org.uk/) |
| Hans Hoffman | Pompeii 1959 | Oil on canvas | [www.tate.org.uk](http://www.tate.org.uk/) |
| Hans Hoffman | Nulli Secundus  1964 | Oil on canvas | [www.tate.org.uk](http://www.tate.org.uk/) |
| Edward Hopper | Night Windows 1928 | Oil on canvas | [www.moma.org](http://www.tate.org.uk/) |
| Edward Hopper | Gas 1940 | Oil on canvas | [www.moma.org](http://www.tate.org.uk/) |
| Jorg Immendorf | Café Deutschland 1978 | Gouache on paper | [www.moma.org](http://www.tate.org.uk/) |
| Augustus John OM | Robin circa 1912 | Oil on wood | [www.tate.org.uk](http://www.tate.org.uk/) |
| Augustus John OM | The Little Railway, Martigues  1928 | Oil on canvas | [www.tate.org.uk](http://www.tate.org.uk/) |
| Augustus John OM | Lyric Fantasy  circa 1913-4 | Oil and pencil on canvas | [www.tate.org.uk](http://www.tate.org.uk/) |
| Augustus John OM | Blue Cineraria  circa 1928 | Oil on canvas | [www.tate.org.uk](http://www.tate.org.uk/) |
| Augustus John OM | Woman Smiling  1908-9 | Oil on canvas | [www.tate.org.uk](http://www.tate.org.uk/) |
| Jasper Johns | Green Target 1955 | Encaustic on newspaper and cloth over canvas, | [www.moma.org](http://www.tate.org.uk/) |
| Jasper Johns | 0 through 9 1961 | Oil on canvas | [www.tate.org.uk](http://www.tate.org.uk/) |
| Jasper Johns | Dancers on a Plane 1980-81 | Oil on canvas and bronze frame | [www.tate.org.uk](http://www.tate.org.uk/) |
| Donald Judd | from Untitled (P77496-P77505; complete) no title 1988 | Woodcut on paper | [www.moma.org](http://www.tate.org.uk/) |
| Donald Judd | Untitled 1961 | Synthetic polymer paint and sand on composition board, | [www.moma.org](http://www.tate.org.uk/) |
| Donald Judd | Untitled 1961_9 | Woodcut on paper | [www.tate.org.uk](http://www.tate.org.uk/) |
| Donald Judd | from Untitled (P11522-P11531; complete) (no title)1992-3 | Woodcut on paper | [www.tate.org.uk](http://www.tate.org.uk/) |
| Frieda Kahlo | Self Portrait With Cropped Hair 1940 | Oil on canvas | [www.moma.org](http://www.tate.org.uk/) |
| Frida Kahlo | My Grandparents, My Parents, and I (Family Tree) 1936 | Oil and tempera on zinc | [www.moma.org](http://www.tate.org.uk/) |
| Wassily Kandinsky | Swinging 1925 | Oil on board | [www.tate.org.uk](http://www.tate.org.uk/) |
| Wassily Kandinsky | Lake Starnberg  1908 | Oil on board | [www.tate.org.uk](http://www.tate.org.uk/) |
| Anish Kapoor | Untitled 1987 | Gouache on paper | [www.tate.org.uk](http://www.tate.org.uk/) |
| Anish Kapoor | from Blackness from Her Womb (P78608-P78620) [no title] 2000 | Etching on paper on paper | [www.tate.org.uk](http://www.tate.org.uk/) |
| Alex Katz | Night Branch  1994 | Oil on board | [www.tate.org.uk](http://www.tate.org.uk/) |
| Alex Katz | Pansies  1967 | Oil on board | [www.tate.org.uk](http://www.tate.org.uk/) |
| Alex Katz | East Window  1979 | Oil on board | [www.tate.org.uk](http://www.tate.org.uk/) |
| Ellsworth Kelly | Black Square with Blue  1970 | Oil on canvas | [www.tate.org.uk](http://www.tate.org.uk/) |
| Ellsworth Kelly | Orange Relief with Green  1991 | Oil on canvas | [www.tate.org.uk](http://www.tate.org.uk/) |
| Ellsworth Kelly | Méditerannée  1952 | Oil on wood | [www.tate.org.uk](http://www.tate.org.uk/) |
| Anselm Kieffer | Palette  1981 | Oil, shellac and emulsion on canvas | [www.tate.org.uk](http://www.tate.org.uk/) |
| Martin Kippenberger | Event Poster S.O. 36  1979 | Lithograph on paper | [www.moma.org](http://www.tate.org.uk/) |
| Martin Kippenberger | War is no Nice 1985 | Oil and silicone rubber on canvas | [www.moma.org](http://www.tate.org.uk/) |
| Paul Klee | They're Biting  1920 | Drawing and oil on paper | [www.moma.org](http://www.tate.org.uk/) |
| Paul Klee | Drinker c 1909 | Etching and drypoint | [www.moma.org](http://www.tate.org.uk/) |
| Paul Klee | A Young Lady's Adventure  1922 | Watercolour on paper | [www.moma.org](http://www.tate.org.uk/) |
| Paul Klee | The Protector 1926 | Pen and ink on paper on board | www.tate.org.uk |
| Yves Klein | Blue Monochrome, 1961 | Dry pigment in synthetic polymer medium on cotton over plywood, | www.moma.org |
| Yves Klein | IKB 79 1959 | Paint on canvas on wood | [www.tate.org.uk](http://www.moma.org/) |
| Gustav Klimt | Hope, II 1907-08 | Oil, gold and platinum on canvas | [www.moma.org](http://www.moma.org/) |
| Gustav Klimt | The Park 1910 | Oil on canvas | [www.moma.org](http://www.moma.org/) |
| Gustav Klimt | Schwangere mit Mann nach links (Pregnant Woman with Man)about 1903 - 1904 | Black chalk on paper | [www.nationalgalleries.org](http://www.moma.org/) |
| Franz Kline | Meryon 1960 -61 | oil on canvas | www.tate.org.uk |
| Jeff Koons | Art Magazine Ads  1988-9 | Lithograph on paper | www.tate.org.uk |
| Jannis Kounellis | from Kounellis 99 (P78423-P78434; complete) [no title] 1999 | Etching on paper | [www.tate.org.uk](http://www.moma.org/) |
| Fernand Leger | Still Life with a Beer Mug  1921-2 | oil on canvas | [www.tate.org.uk](http://www.moma.org/) |
| Fernand Leger | Three Bottles  1954 | Oil on canvas | [www.tate.org.uk](http://www.moma.org/) |
| Fernand Leger | Leaves and Shell  1927 | Oil on canvas | www.tate.org.uk |
| Sol LeWitt | Arcs from Four Corners  1986 | Woodcut on paper | [www.tate.org.uk](http://www.moma.org/) |
| Sol LeWitt | A Square Divided Horizontally and Vertically into Four Equal Parts, Each with a Different Direction of Alternating Parallel Bands of Lines  1982 | Watercolour and relief print on paper | [www.tate.org.uk](http://www.moma.org/) |
| Roy Lichtenstein | Landscape 5 from Ten Landscapes, 1967 | One from a portfolio of ten screenprints, composition and sheet | [www.moma.org](http://www.moma.org/) |
| Roy Lichtenstein | Moonscape 1965 | Screenprint on plastic | [www.tate.org.uk](http://www.nationalgalleries.org/) |
| L S Lowry | Hillside in Wales  1962 | Oil on canvas | [www.tate.org.uk](http://www.nationalgalleries.org/) |
| L S Lowry | A Young Man 1955 | Oil on canvas | www.tate.org.uk |
| L S Lowry | Coming out of School 1927 | Oil on wood | www.tate.org.uk |
| Sarah Lucas | from Self-Portraits 1990-1998 (P78443-P78454; complete) Self Portrait with Mug of Tea 1993 | Inkjet print on paper | www.tate.org.uk |
| Sarah Lucas | from Self-Portraits 1990-1998 (P78443-P78454; complete) Human Toilet Revisited 1998 | Inkjet print on paper | www.tate.org.uk |
| Sarah Lucas | Sod You Gits  1991 | Photograph on paper | www.tate.org.uk |
| Rene Magritte | Man with a Newspaper 1928 | Oil on canvas | www.tate.org.uk |
| Rene Magritte | The Reckless Sleeper 1928 | Oil on canvas | www.tate.org.uk |
| Kasimir Malevich | Dynamic Suprematism 1915 or 16 | oil on canvas | www.tate.org.uk |
| Robert Mangold | Red Wall  1965 | Oil on Masonite | www.tate.org.uk |
| Man Ray | Untitled 1969 | Lithograph and screenprint on paper | www.tate.org.uk |
| Piero Manzoni | Achrome  1958 | China-clay on canvas | www.tate.org.uk |
| Brice Marden | Han Shan Exit  1992 | Etching and sugarlift aquatint on paper | www.tate.org.uk |
| Brice Marden | Couplet III  1988-9 | Oil on canvas | www.tate.org.uk |
| Brice Marden | Untitled  1973-9 | Etching on paper | www.tate.org.uk |
| Agnes Martin | Happy Holiday  1999 | Acrylic and graphite on canvas | www.tate.org.uk |
| Henri Matisse | The Snail 1953 | Gouache on paper, cut and pasted on paper mounted on canvas | www.tate.org.uk |
| Henri Matisse | Reading Woman with a Parasol 1921 | Oil on canvas | www.tate.org.uk |
| Henri Matisse | Draped Nude 1936 | Oil on canvas | www.tate.org.uk |
| Joan Miro | Message froma Friend 1964 | Oil on canvas | www.tate.org.uk |
| Amadeo Modigliani | Portrait of a Girl c 1917 | Oil on canvas | www.tate.org.uk |
| Amadeo Modigliani | The Little Peasant c 1918 | Oil on canvas | www.tate.org.uk |
| Amedeo Modigliani | Caryatid with a Vase c 1914 | Watercolour on paper | www.tate.org.uk |
| Piet Mondrian | Broadway Boogie Woogie | Oil on canvas | www.moma.org |
| Piet Mondrian | Composition with Red, Blue, Black, Yellow, and Gray | Oil on canvas | www.moma.org |
| Piet Mondrian | Tableau I: Lozenge with Four Lines and Gray 1926 | Oil on canvas | www.moma.org |
| Piet Mondrian | Composition with Double Line and Yellow, 1932 | Oil on canvas | [www.nationalgalleries.org](http://www.nationalgalleries.org/) |
| Claude Monet | The Seine at Port-Villez  1894 | Oil on canvas | [www.tate.org.uk](http://www.tate.org.uk/) |
| Claude Monet | Woman Seated on a Bench c 1874 | Oil on canvas | www.tate.org.uk |
| Henry Moore | The Artist's Sister Mary 1926 | Pen and ink and ink wash on paper | www.tate.org.uk |
| Giorgio Morandi | Still Life 1946 | Oil on canvas | www.tate.org.uk |
| Robert Morris | Blind Time XIII 1973 | Graphite on paper | www.moma.org |
| Robert Motherwell | Game of Chance  1987 | Lithograph, aquatint, collage, pastel and acrylic on paper | www.tate.org.uk |
| Robert Motherwell | Ulysses  1947 | Oil and cardboard on wood | www.tate.org.uk |
| Robert Motherwell | Open No. 122 in Scarlet and Blue  1969 | Acrylic and drawing on canvas | www.tate.org.uk |
| Edvard Munch | The Sick Child 1907 | Oil on canvas | www.tate.org.uk |
| Laszlo Moholy-Nagy | K VII  1922 | Oil on canvas | www.tate.org.uk |
| Bruce Nauman | Face mask 1981 | Synthetic polymer paint, charcoal, and pencil on paper, | www.moma.org |
| Bruce Nauman | Raw-War 1971 | Lithograph on Paper | www.tate.org.uk |
| Bruce Nauman | *from* Studies for Holograms (a-e) (P77629-P77633; complete) a 1970 | Screenprint on paper | www.moma.org |
| Barnett Newman | Canto IX 1963-4 | Lithograph on paper | www.tate.org.uk |
| Barnett Newman | Adam 1951-2 | Oil on canvas | www.tate.org.uk |
| Barnett Newman | Moment 1946 | Oil on canvas | www.tate.org.uk |
| Ben Nicholson | Feb 28-53 (vertical seconds)  1953 | Oil on canvas | www.tate.org.uk |
| Hermann Nitsch | Poured Painting  1963 | Oil on canvas | www.tate.org.uk |
| Hermann Nitsch | Blood Picture  1962 | Mixed media on canvas | www.tate.org.uk |
| Georgia O'Keeffee | Lake George, Coat and Red, 1919 | Oil on canvas | www.moma.org |
| Claus Oldenberg | Notes (Micky Mouse)  1968 | Lithograph on paper | www.tate.org.uk |
| Eduardo Paolozzi | Inkwells Gold  1962 | Screenprint on paper | www.tate.org.uk |
| Francis Picabia | Otaïti  1930 | Oil and resin on canvas | www.tate.org.uk |
| Francis Picabia | Portrait of a Doctor  circa 1935-8 | Oil on canvas | www.tate.org.uk |
| Pablo Picasso | Bullfight Scene  1960 | Brush and ink on paper | [www.tate.org.uk](http://www.tate.org.uk/) |
| Pablo Picasso | Seated Woman in a Chemise  1923 | Oil on canvas | [www.tate.org.uk](http://www.tate.org.uk/) |
| Pablo Picasso | Head of a Young Boy 1945 | Lithograph on paper | www.tate.org.uk |
| Pablo Picasso | Weeping Woman 1937 | Oil on canvas | www.tate.org.uk |
| Jackson Pollock | Naked Man with Knife  circa 1938-40 | Oil on canvas | [www.tate.org.uk](http://www.tate.org.uk/) |
| Jackson Pollock | Landscape with Steer 1936-37 | Lithograph with airbrushed enamel additions, | www.moma.org |
| Jackson Pollock | Yellow Islands  1952 | Oil on canvas | [www.tate.org.uk](http://www.tate.org.uk/) |
| Richard Prince | Untitled 1999 | Synthetic polymer paint and silkscreened ink on paper, | www.moma.org |
| Arnulf Rainer | Untitled (Death Mask)  1978 | Oil, pastel and photograph on paper | www.tate.org.uk |
| Robert Rauschenberg | The Razorback Bunch (Etching I)  1980 | Intaglio print on paper | www.moma.org |
| Odile Redon | Profile of a Woman with a Vase of Flowers  circa 1895-1905 | Oil on canvas | www.tate.org.uk |
| Paula Rego | War  2005 | Pastel on paper on aluminium | www.tate.org.uk |
| Paula Rego | The Dance 1988 | Acrylic on paper laid on canvas | www.tate.org.uk |
| Paula Rego | from Pendle Witches (P77902-P77913; complete) Moth 1996 | Etching and aquatint on paper | www.tate.org.uk |
| Ad Reinhardt | Abstract Painting No. 5  1962 | Oil on canvas | www.tate.org.uk |
| Ad Reinhardt | Abstract Painting  circa 1951-2 | Oil on canvas | www.tate.org.uk |
| Pierre Auguste Renoir | Pinning the Hat, 1897 | Lithograph | www.moma.org |
| August Renoir | Nude on a Couch 1945 | Oil on canvas | www.tate.org.uk |
| August Renoir | Head of a Girl 1898 | Oil on canvas | www.tate.org.uk |
| August Renoir | Peaches and Almonds 1901 | Oil on canvas | www.tate.org.uk |
| Gerhard Richter | Abstract Painting (809-3)  1994 | Oil on canvas | www.tate.org.uk |
| Gerhard Richter | Abstract Painting (Silicate) (880-4)  2002 | Oil on Alu-Dibond | www.tate.org.uk |
| Bridget Riley | Achæan  1981 | Oil on canvas | www.tate.org.uk |
| Bridget Riley | Hesitate 1964 | Oil on canvas | www.tate.org.uk |
| Bridget Riley | Nataraja  1993 | Oil on canvas | www.tate.org.uk |
| Bridget Riley | Deny II  1967 | PVA emulsion on canvas | www.tate.org.uk |
| Diego Rivera | Agrarian Leader Zapata 1931 | Fresco, | www.moma.org |
| Alexsandr Rodchenko | Untitled 1929 | Gelatin silver print, | www.moma.org |
| James Rosenquist | from Leo Castelli's 90th Birthday Portfolio (L02354-L02362; complete) The Flame Dances on Leo's Book 1997 | Lithograph on paper | www.tate.org.uk |
| James Rosenquist | Off the Continental Divide  1973-4 | Lithograph on paper | [www.tate.org.uk](http://www.moma.org/) |
| Marc Rothko | No.5/No.22, 1950 | Oil on canvas | [www.moma.org](http://www.moma.org/) |
| Mark Rothko | Light Red over Black 1957 | oil on canvas | [www.tate.org.uk](http://www.moma.org/) |
| Mark Rothko | Red on Maroon 1959 | Oil on canvas | [www.tate.org.uk](http://www.moma.org/) |
| Mark Rothko | Untitled c 1950-2 | Oil on canvas | [www.tate.org.uk](http://www.moma.org/) |
| Georges Roualt | The Three Judges  circa 1936 | Oil on board laid on canvas | [www.tate.org.uk](http://www.moma.org/) |
| Henri Rousseau | Bouquet of Flowers c 1909-10 | Oil on canvas | [www.tate.org.uk](http://www.moma.org/) |
| Edward Ruscha | Time Is Up  1989 | Lithograph on paper | [www.tate.org.uk](http://www.moma.org/) |
| David Salle | Muscular Paper 1985 | Oil, synthetic polymer paint, and charcoal on canvas and fabric, with painted wood, in three parts, | [www.moma.org](http://www.moma.org/) |
| David Salle | from High and Low (P12243-P12247) Fast and Slow 1994 | Lithograph and woodcut on paper | [www.tate.org.uk](http://www.moma.org/) |
| August Sander | Sisters 1927 | Gelatin silver print, | [www.moma.org](http://www.moma.org/) |
| Egon Schiele | Girl putting on a Shoe 1910 | Watercolour and charcoal on paper | [www.moma.org](http://www.moma.org/) |
| Egon Schiele | Sorrow 1914 | Drypoint | [www.moma.org](http://www.moma.org/) |
| Richard Serra | Hreppholar I from Hreppholar I-VII, 1999 | Etching | [www.moma.org](http://www.moma.org/) |
| Richard Serra | Screech  1996 | Etching on paper | [www.tate.org.uk](http://www.moma.org/) |
| Richard Serra | from Leo Castelli's 90th Birthday Portfolio (L02354-L02362; complete) Leo 1997 | Etching on paper | [www.tate.org.uk](http://www.moma.org/) |
| Cindy Sherman | Untitled #99 1982 | Photograph on paper | [www.tate.org.uk](http://www.moma.org/) |
| Cindy Sherman | Untitled A  1975 | Photograph on paper | [www.tate.org.uk](http://www.moma.org/) |
| Cindy Sherman | Untitled Film Still #48  1979, reprinted 1998 | Photograph on paper | [www.tate.org.uk](http://www.moma.org/) |
| Cindy Sherman | Untitled  1976, printed 2000 | Photograph on paper | [www.tate.org.uk](http://www.moma.org/) |
| David Smith | Painting 1964 1964 | Oil on canvas laid on wood | [www.tate.org.uk](http://www.moma.org/) |
| Robert Smithson | Ithaca Mirror Trail, Ithaca, New York  1969 | Mixed media | [www.tate.org.uk](http://www.moma.org/) |
| Chaim Soutine | The Road up the Hill c1924 | Oil on canvas | [www.tate.org.uk](http://www.moma.org/) |
| Chaim Soutine | Landscape at Ceret c1920-1 | Oil on canvas | [www.tate.org.uk](http://www.moma.org/) |
| Chaim Soutine | Cagnes Landscape with Tree 1925-6 | Oil on canvas | [www.tate.org.uk](http://www.moma.org/) |
| Stanley Spencer | Zacharias and Elizabeth  1913-14 | Oil and pencil on canvas | [www.tate.org.uk](http://www.moma.org/) |
| Stanley Spencer | The Centurion's Servant  1914 | Oil on canvas | [www.tate.org.uk](http://www.moma.org/) |
| Stanley Spencer | Dinner on the Hotel Lawn 1956-7 | Oil on canvas | [www.tate.org.uk](http://www.moma.org/) |
| Stanley Spencer | The Roundabout 1923 | Oil on canvas | [www.tate.org.uk](http://www.moma.org/) |
| Nicolas de Stael | Marathon  1948 | Oil on canvas | [www.tate.org.uk](http://www.moma.org/) |
| Nicolas de Stael | Landscape Study  1952 | Oil on board | [www.tate.org.uk](http://www.moma.org/) |
| Nicolas de Stael | Composition 1950  1950 | Oil on board | [www.tate.org.uk](http://www.moma.org/) |
| Alfred Stieglitz | The Steerage 1907 | Photogravure | [www.nationalgalleries.org](http://www.moma.org/) |
| Antoni Tapies | Grey Ochre  1958 | Oil, epoxy resin and marble dust on canvas | www.tate.org.uk |
| Jean Tinguely | Chaos I  1972 | Intaglio print on paper | [www.tate.org.uk](http://www.moma.org/) |
| Cy Twombley | from Quattro Stagioni (A Painting in Four Parts) (T07887-T07890; complete). Quattro Stagioni: Primavera 1993-5 | Acrylic, oil, crayon, and pencil on canvas | [www.tate.org.uk](http://www.moma.org/) |
| Cy Twombley | from Quattro Stagioni (A Painting in Four Parts) (T07887-T07890; complete). Quattro Stagioni: Estate 1993-5 | Acrylic, oil, crayon, and pencil on canvas | [www.tate.org.uk](http://www.moma.org/) |
| Cy Twombly | Untitled, 1970 | Oil-based house paint and crayon on canvas, | [www.moma.org](http://www.moma.org/) |
| Edouard Vuillard | Interior, Mother and Sister of the Artist, 1893 | Oil on canvas | [www.moma.org](http://www.moma.org/) |
| Edouard Vuillard | Sunlit Interior c 1920 | Oil on canvas | [www.tate.org.uk](http://www.moma.org/) |
| Edouard Vuillard | Landscape_House on the Left 1900 | Oil on canvas | [www.tate.org.uk](http://www.moma.org/) |
| Edouard Vuillard | Girl in an Interior c1910 | Oil on canvas | [www.tate.org.uk](http://www.moma.org/) |
| Edouard Vuillard | The Laden Table c 1908 | Oil on canvas | [www.tate.org.uk](http://www.moma.org/) |
| Mark Wallinger | Half-Brother (Exit to Nowhere - Machiavellian)  1994-5 | Oil on canvas | [www.tate.org.uk](http://www.moma.org/) |
| Mark Wallinger | From Bugs: A Portfolio (P78511-P78520; complete) King Edward and the Colorado Beetle 2000 | Potato print on paper | [www.tate.org.uk](http://www.moma.org/) |
| Mark Wallinger | Where There's Muck  1985 | Mixed media | [www.tate.org.uk](http://www.moma.org/) |
| Andy Warhol | Camouflage 1987 | Portfolio of eight screen prints | [www.moma.org](http://www.moma.org/) |
| Andy Warhol | [from Mao Tse-Tung, [no title] 1972](http://www.tate.org.uk/servlet/ViewWork?cgroupid=999999961&workid=15971&searchid=10382) | Screenprint on paper | [www.tate.org.uk](http://www.moma.org/) |
| Andy Warhol | Birmingham Race Riot  1964 | Screenprint on paper | [www.tate.org.uk](http://www.tate.org.uk/) |
| Andy Warhol | From Marilyn (P07121-P07130; complete) [no title] 1967 | Screenprint on paper | www.tate.org.uk |
| Andy Warhol | Marilyn Diptych  1962 | Acrylic on canvas | www.tate.org.uk |
| Weegee | Woman Shot froma Canon, New york | Gelatin silver print | [www.moma.org](http://www.moma.org/) |
| Weegee | Victory Celebration 1945 | Gelatin silver print, | www.moma.org |
| David Hockney | Hawthorne Blossom near Rudston, 2008 | Oil on two canvases | www.**hockney**pictures.com |
| David Hockney | Piscine a Minuit (paper pool 19), 1978 | Coloured and pressed paper pulp | www.**hockney**pictures.com |
