# Supplementary material for: Art expertise modulates the emotional response to modern art, especially abstract: an ERP investigation
Source: Front Hum Neurosci. 2015 Sep 30;9:525. doi: 10.3389/fnhum.2015.00525 (PMC4876367; doi:10.3389/fnhum.2015.00525)
Supplement: Supplementary file 2 [file DataSheet_2.DOCX]

**Table 5a.** Showing the significant main effects of a 2 (Group: NA, A) x 2 (Stimuli: FSS, RSS) x 3 (Hemisphere: L, M, R) x 4 (Location: F, P, C, O) analysis of variance (ANOVA) to interpret EEG responses to simple stimuli. In instances where the data failed the Mauchly’s W sphericity test (p < .05) the Greehouse-Geisser test was substituted. All pairwise comparisons were Bonferroni adjusted, the mean difference was significant to .05 level. *(blue italics = nearly significant, would be significant if Greenhouse-Geisser test not applied)*

| **Interaction** | **P1 amplitude** | **P1 latency** | **N1 amplitude** | **N1 latency** | **P2 amplitude** | **P2 latency** | **N2 amplitude** | **N2 latency** | **P3 amplitude** | **P3 latency** |
| --- | --- | --- | --- | --- | --- | --- | --- | --- | --- | --- |
| **Stimuli** |  | *F* (1, 29) = 18.14, p < .001, ŋ²p = .39 | *F* ( 1,29) = 26.73, p < .001, ŋ²p = .48 |  |  | *F* (1,29) = 5.76, p < .05, ŋ²p = .17 | *F* (1,29) = 33.19, p < .001, ŋ²p = .53 |  |  |  |
| **Stimuli x Group** |  |  |  |  |  |  |  |  |  |  |
| **Hemisphere** |  |  | *F* (2, 58) = 8.61, p < .005, ŋ²p = .23 | *F* (1.47, 42.59) = 14.06, p < .001, ŋ²p = .33 |  | *F* (1.72, 49.81) = 10.34, p < .001, ŋ²p = .26 | *F* (2,58) = 6.33, p < .005, ŋ²p = .18 | *F* (1.22, 35.32) = 6.86, p < .01, ŋ²p = .19 |  | *F* (1.36, 39.32) = 6.18, p < .05, ŋ²p = .18 |
| **Hemisphere x Group** |  |  |  |  |  |  |  |  |  |  |
| **Location** |  | *F* (1.77, 51.18) = 3.70, p < .05, ŋ²p = .11 | *F* (2.12, 61.41) = 13.24, p< .001, ŋ²p = .31 | *F*( 2.10, 60.96) = 5.00, p < .01, ŋ²p = .15 | *F* (1.59, 46.23) = 38.60, p < .001, ŋ²p = .57 |  | *F* (1.78, 51.60) = 28.86, p < .001, ŋ²p = .50 | *F* (1.72, 49.79) = 3.84, p < .05, ŋ²p = .12 | *F* (2.04, 59.12) = 21.34, p < .001, ŋ²p = .42 | *F* (2.53, 73.36) = 5.18, p < .005, ŋ²p = .15 |
| **Location x Group** |  |  | *F* (2.12, 61.41) = 4.31, p < .05, ŋ²p = .13 |  | *F* (1.59, 46.23) = 5.55, p < .05, ŋ²p = .16 |  | *F* (1.78, 51.60) = 3.88, p , .05, ŋ²p = .12 |  | *F* (2.04, 59.12) = 11.21, p < .001, ŋ²p = .28 |  |
| **Stimuli x Hemisphere** | *F* (2, 58) = 9.2, p <.001, ŋ²p = .24 |  | *F* (1.88, 54.37) = 23.94, p < .001, ŋ²p = .45 |  | *F* (2, 58) = 6.10, p < .005, ŋ²p = .17 |  | *F* (1.6, 46.39) = 5.19, p < .05, ŋ²p = .15 |  |  |  |
| **Stimuli x Hemisphere x Group** |  |  |  |  |  |  |  |  |  |  |

| **Interaction** | **P1 amplitude** | **P1 latency** | **N1 amplitude** | **N1 latency** | **P2 amplitude** | **P2 latency** | **N2 amplitude** | **N2 latency** | **P3 amplitude** | **P3 latency** |
| --- | --- | --- | --- | --- | --- | --- | --- | --- | --- | --- |
| **Stimuli x Location** | *F* (2.02, 58.62) = 6.15, p < .005, ŋ²p = .18 | *F* (2.01, 58.30) = 9.55, p < .001, ŋ²p = .25 | *F* (1.77, 51.41) = 46.27, p < .001, ŋ²p = .62 | *F* (2.34, 67.92) = 10.65, p < .001, ŋ²p = .27 | *F* (1.78, 51. 70) = 48.84, p < .001, ŋ²p = .63 | *F(2.10, 60.82) = 2.9, p = .06*, ŋ²p = .09 | *F* (1.88, 54.58) = 42.91, p < .001, ŋ²p = .60 | *F* (1.61, 46.63) = 6.65, p < .01, ŋ²p = .19 |  |  |
| **Stimuli x Location x Group** |  |  |  |  |  |  |  |  |  |  |
| **Hemisphere x Location** | *F* (2.63, 76.12) = 3.04, p < .05, ŋ²p = .10 | *F* (2.96, 85.85) = 3.58, p < .05, ŋ²p = .11 | *F* (4.25, 123.22) = 3.68, P < .01, ŋ²p = .11 |  | *F* (3.55, 102.79) = 6.79, p < .001, ŋ²p = .19 | *F (3.26, 94.59) = 2.25, p = .08, ŋ²p = .07* | *F* (3.69, 107.08), = 3.05, p < .05, ŋ²p = .10 | *F* (2.99, 86.80) = 4.34, p < .01, ŋ²p = .13 | *F* (3.78, 109.52) = 3.95, p , .01, ŋ²p = .12 | *F* (2.97, 86.23) = 3.48, p < .05, ŋ²p = .11 |
| **Hemisphere x Location x Group** |  | *F* (2.96, 85.85) = 3.25, p < .05, ŋ²p = .10 |  | *F* (2.69, 77.83) = 2.77, p < .05, ŋ²p = .09 |  | *F (3.26, 94.59) = 2.53, p = .06, ŋ²p = .08* | *F* (3.69, 107.08) = 3.14, p < .001, ŋ²p = .18 | *F* (2.99, 86.80) = 2.86, p < .05, ŋ²p = .09 |  | *F* (2.97, 86.23) = 3.4, p < .05, ŋ²p = .11 |
| **Stimuli x Hemisphere x Location** | *F* (3.11, 90.31) = 3.95, P < .05, ŋ²p = .39 |  | *F* (4.16, 120.63) = 5.52, p < .001, ŋ²p = .16 |  | *F* (3.87, 112.29) = 11.79, p < .001, ŋ²p=.29 |  | *F* (6, 174) = 6.43, p < .001, ŋ²p=.18 |  |  |  |
| **Stimuli x Hemisphere x Location x Group** |  |  |  |  |  |  |  |  |  |  |
|  |  |  |  |  |  |  |  |  |  |  |
| **Pairwise comparisons:** | **P1 amplitude** | **P1 latency** | **N1 amplitude** | **N1 latency** | **P2 amplitude** | **P2 latency** | **N2 amplitude** | **N2 latency** | **P3 amplitude** | **P3 latency** |
| **Group** |  |  |  |  |  |  |  |  |  |  |
| **Stimuli** |  |  | RSS >FSS, p <.001 |  |  | FSS>RSS, p<.05 | FSS <RSS, p<.001 |  |  |  |
| **Hemisphere** |  |  | M>L, p<.01  M>R, p<.005 | M>R, p<.001  M>L, p<.05  R>L p<.05 |  | L<M, p<.005  L<R, p<.05 | L<M, p<.005 | L<R, p<.01  M<R, p<.05 |  | L<M, p<.05  L<R, p<.05 |
| **Location** |  | F>P, p < .001 | F>P, p<.005  F>O, p<.005  C>P, p<.001  C>O, p<.005 | C>P, p<.05  C>O, p<.05 | F<P, p<.001  F<O, p<.001  C<P, p<.001  C<O, p<.001  P<O, p<.05 |  | F>P, p<.05  F>O, p<.001  C>P, p<.001  C>O, p<.001  P>O, p<.001 | P>F, p< .01 | F<C, p<.05  F<P, p<.001  F<O, p<.01  C<P, p<.001  C<O, p<.05  P<O, p<.001 | C>O, p<.05 |

*Non-Target Stimuli*

Under free viewing conditions of the non-target stimuli the mean amplitude of the 5 ERPs of interest (the P1, N1, P2, N2 and P3) was larger in response to RSS than to FSS, and the latency was longer for the FSS. An early positivity was evident at occipital sites in response to both stimuli, with larger amplitude and shorter latency in response to RSS than for FSS, particularly for the artists. The P1 and N1 waves are obligatory, exogenous sensory responses, known to be larger for attended than non-attended stimuli, which vary in amplitude and latency according to low level physical characteristics of the stimuli (Hillyard & Anllo-Vento, 1998; Luck & Kappenham, 2012; O’Donnell et al., 2012). The anterior N2 effect is observed only when subjects are searching for an item that differs from the rest of the array (Luck, 2012), and indexes selective attention (Olofsson & Polich, 2007). Here the amplitude of the N2 component was larger for the RSS at frontocentral sites. The P3 amplitude is also influenced by the amount of attention allocated to a stimulus (Luck & Kappenham, 2012; Polich 2007a) and here the increased amplitude in response to RSS at occipito-parietal sites suggests the attention and visual arousal of both groups increased, despite the fact that no response was required to either of these stimuli. The consistently longer latency in response to the FSS may be attributed to difference in the brightness or change in colour between the non-target stimuli (Kappenham & Luck, 2012). These findings suggest that the RSS recruited more attentional resources and selective attention than did the FSS, and that this effect was enhanced in artists.

**Table 5b.** Showing the significant main effects of a 2 (Group: NA, A) x 3 (Art Stimuli: AA, RA, IA) x 3 (Hemisphere: L, M, R) x 4 (Location: F, P, C, O) analysis of variance (ANOVA) to interpret EEG responses to target stimuli. In instances where the data failed the Mauchly’s W sphericity test (p < .05) the Greehouse-Geisser test was substituted. All pairwise comparisons were Bonferroni adjusted, the mean difference was significant to .05 level. *(blue italics = nearly significant, would be significant if Greenhouse-Geisser test not applied)*

| **Interaction** | **P1 amplitude** | **P1 latency** | **N1 amplitude** | **N1 latency** | **P2 amplitude** | **P2 latency** | **N2 amplitude** | **N2 latency** | **P3 amplitude** | **P3 latency** | **LPC amplitude** |
| --- | --- | --- | --- | --- | --- | --- | --- | --- | --- | --- | --- |
| **Art Stimuli** | *F* (2, 58) = 23.67, p < .001, ŋ²p = .45 | *F* (2, 58) = 27.61, p < .001, ŋ²p = .49 |  | *F* (1.34, 38.78) = 231.71, p < .001, ŋ²p = .89 | *F* (2, 58) = 23.1, p < .001, ŋ²p = .44 | *F* (1.37, 39.70) = 178.98, p < .001, ŋ²p = .86 | *F* (1.64, 47.64) = 12.79, p < .001, ŋ²p = .31 | *F* (1.19, 34.44) = 596.27, p < .001, ŋ²p = .95 |  | *F* (1.5, 43.7) = 12.41, p < .001, ŋ²p = .30 |  |
| **Art Stimuli x Group** |  |  |  |  | *F* (1.97, 57.04) = 4.08, p ¸.05, ŋ²p = .12 |  |  |  |  |  |  |
| **Hemisphere** |  | *F* (1.39, 40.16) = 3.14, p=.07, , ŋ²p = .10 | *F* (2, 58) = 15.86, p < .001, ŋ²p = .35 | *F* (1.34, 38.78) = 9.74, p < .005, ŋ²p = .25 |  | *F* (2, 58) = 7.18, P < .005, ŋ²p = .20 | *F* (2, 58) = .19.41, p < .001, ŋ²p = .40 | *F* (1.41, 40.82) = 12.64, p < .001, ŋ²p = .30 |  | *F* (1.38, 39.98) = 9.17, p < .005, ŋ²p = .24 | *F* (2,58) = 5.70, p<.001, , ŋ²p = .56 |
| **Hemisphere x Group** |  |  |  |  |  |  |  |  |  |  |  |
| **Location** | *F* (3, 87) = 23.80, p < .001, ŋ²p = .45 | *F* (1.74, 50.38) = 12.33, p < .001, ŋ²p = .30 | *F* (3, 87) = 6.26, p < .005, ŋ²p = .18 |  | *F* (1.91, 55.48)= 41.74, p < .001, ŋ²p = .59 | *F* (1.98, 57.31) = 5.18, p < .01, ŋ²p =.15 | *F* (1.78, 51.56) = 77.90, p < .001, ŋ²p = .73 |  | *F* (2.18, 63.25) = 45.97, p < .001, ŋ²p = .61 | *F* (92.05, 59.43) = 5.43, p < .01, ŋ²p = .16 | *F* (3, 87) = 36.67, p <.001, ŋ²p = .56 |
| **Location x Group** | *F* (1.98, 57.28) = 4.71, p < .05, ŋ²p = .14 |  | *F* (3, 87) = 4.58, p < .01, ŋ²p = .14 |  | *F* (1.91, 55.48) = 7.07, p < .005, ŋ²p = .20 |  | *F* (1.78, 51.56) = 5.43, p < .01, ŋ²p = .16 |  | *F* (2.18, 63.25) = 3.64, p < .05, ŋ²p = .11 |  | *F (1.97, 57.16) = 2.90, p = .06, ŋ²p =.09* |
| **Art Stimuli x Hemisphere** |  |  | *F* (4, 116) = 3.38, p < .01, ŋ²p = .10 | *F* (3.11, 90.14) = 8.25, p < .001, ŋ²p = .22 |  |  | *F* (3.13, 90.70) = 3.23, p < .05, ŋ²p = .10 | *F* (2.72, 78.88) = 6.01, p <.005, ŋ²p = .17 | *F* (2.60, 75.38) = 3.38, p < .05, ŋ²p = .10 |  |  |
| **Interaction** | **P1 amplitude** | **P1 latency** | **N1 amplitude** | **N1 latency** | **P2 amplitude** | **P2 latency** | **N2 amplitude** | **N2 latency** | **P3 amplitude** | **P3 latency** | **LPC amplitude** |
| **Art Stimuli x Hemisphere x Group** |  |  |  |  |  |  |  |  |  |  |  |
| **Art Stimuli x Location** | *F* (2.73, 79.28) = 8.94, p < .001, ŋ²p = .24 | *F* (6, 174) = 7.16, p < .001, ŋ²p = .20 |  | *F* (2.46, 71.39) = 7.19, p < .005, ŋ²p = .20 | *F* (3.10, 89.75) = 17.98, p < .001, ŋ²p = .38 | *F* (2.58, 74.95) = 12.59, p< .001, ŋ²p =.30 | *F*(2.04, 59.19), = 43.55, p < .001, ŋ²p = .60 |  | *F* (2.66, 77.12) =15.29, p < .001, ŋ²p = .35 |  | *F* (2.45, 70.95) = 3.46, p < .05, ŋ²p = .11 |
| **Art Stimuli x Location x Group** |  |  |  |  | *F (3.10, 89.75) = 2.65, p = .05, ŋ²p = .08* |  |  |  | *F* (2.66, 77.12) = 4.92, p < .01, ŋ²p = .15 |  | *F (2.45, 70.95) = 2.29, p = .098, ŋ²p = .07* |
| **Hemisphere x Location** | *F* (2.96, 85.74) = 4.13, p<.01, ŋ²p = .13 | *F* (3.36, 97.38) = 3.27, p < .01, ŋ²p = .10 | *F* (6, 174) = 5.43, p < .001, ŋ²p = .16 | *F (2.85, 82.65) = 2.44, p = .07 , ŋ²p = .07* | *F* (3.74, 108.41) = 9.18, p < .001, ŋ²p = .24 | *F* (2.88, 83.51) = 3.20, p < .05, ŋ²p = .10 | *F* (3.21, 93.06) = 4.31, p < .01, ŋ²p =.13 | *F (2.71, 78.65) = 2.62, p=.06, ŋ²p = .08* | *F* (3.09, 89.71) = 8.89, p < .001, ŋ²p =.24 | *F* (2.85, 82.76) = 2.77, p < .05, ŋ²p = .09 | *F* (2.86, 82.89) = 7.32, p < .001, ŋ²p =.20 |
| **Hemisphere x Location x Group** |  | *F (3.36, 97.38) = 2.45, p=.06, ŋ²p = .18* |  | *F* (2.85, 82.65) = 3.36, p < .05, ŋ²p = .10 |  | *F* (2.88, 83.51) = 3.41, p < .05, ŋ²p = .11 |  | *F* (2.71, 78.65) = 3.00, p < .05, ŋ²p = .09 |  | *F* (2.85, 82.76) = 2.95, p < .05, ŋ²p = .09 |  |
| **Art Stimuli x Hemisphere x Location** | *F*(3.88, 112.50) = 5.96, p<.05, ŋ²p = .08 | *F* (12, 348) = 2.42, p < .01, ŋ²p = .08 | *F* (12, 348) = 4.46, p < .001, ŋ²p = .13 | *F (5.76, 167.12) =2.13, p = .056, ŋ²p = .07* |  |  | *F* ( 5.53, 160.49) = 4.27, p < .005, ŋ²p =.13 | *F* (4.54, 131.68) = 3.31, p < .05, ŋ²p = .10 | *F (2.77, 80.43) = 1.90, p=.14 ŋ²p = .06* |  |  |
| **Art Stimuli x Hemisphere x Location x Group** |  |  |  |  |  | *F (6.99, 202.63) = 1.91, p =.07, ŋ²p =.06* |  | *F* (4.54, 131.68) = 2.55, p < .05, ŋ²p = .08 |  |  |  |
|  |  |  |  |  |  |  |  |  |  |  |  |
| **Pairwise comparisons** | **P1 amplitude** | **P1 latency** | **N1 amplitude** | **N1 latency** | **P2 amplitude** | **P2 latency** | **N2 amplitude** | **N2 latency** | **P3 amplitude** | **P3 latency** | **LPC amplitude** |
| **Group** |  |  |  |  | *F* (1, 29) =4.96, p < .05, ŋ²p = .15 |  |  |  |  |  |  |
| **Art Stimuli** | AA>IA p<.001  RA >IA p<.001. | AA>IA  p<.001.  RA>IA  p<.001 |  | IA>AA, p<.001  IA>RA, p<.001 | IA>AA, p<.001  IA>RA, p<.001 | AA>IA, p<.001  AA>RA, p<.05  RA>IA, p<.001 | AA>RA, p<.01  AA>IA, p<.001 | AA>IA, p<.001  RA>IA, p<.001 |  | AA>IA, p<.001  RA>IA, p<.005 |  |
| **Hemisphere** |  |  | L>M, p<.001  R>M, p<.001 | M>L, p<.01  R>L, p<.05 |  | M>L, p<.05  R>L, p<.05 | M>L, p<.001  R>M, p<.005 | M>L, p<.005  R>L, p<.005 |  | M>L, p<.005  R>L, p<.01 |  |
| **Location** | P>F, p<.001  P>C, p<.001  O>F, p<.001  O>C, p<.001 | C>F, p<.005  P>F, p<.001  O>F, p<.005  P>C, p<.005 | F>P, p < .05  C>P, p < .01 |  | C>F, p<.005  P>F, p<.001  P>C, p<.001  O>F, p<.001  O>C, p<.001 | C>F, p<.05  P>F, p<.01  P>C, p<.05 | C>F, p<.05  P>F, p<.001  O>F, p<.001  P>C, p<.001  O>C, p<.001 |  | C>F, p<.001  P>F, p<.001  O>F, p<.005  P>C, p<.001  P>O, p<.001 | C>F, p<.005  C>O, p<.05  P>O, p<.05  P>F, p<.05 |  |
